# The simplicity of protein sequence-function relationships

**DOI:** 10.1101/2023.09.02.556057

**Published:** 2023-09-05

**Authors:** Yeonwoo Park, Brian P.H. Metzger, Joseph W. Thornton

**Affiliations:** a Committee on Genetics, Genomics, and Systems Biology, University of Chicago, Chicago, IL 60637; b Department of Ecology and Evolution, University of Chicago, Chicago, IL 60637; c Department of Human Genetics, University of Chicago, Chicago, IL 60637; d Current affiliation: Center for RNA Research, Seoul National University, Seoul, Republic of Korea 08826; e Current affiliation: Department of Biological Sciences, Purdue University, West Lafayette, IN 47907

**Keywords:** Sequence-function relationship, genetic architecture, epistasis, reference-free analysis

## Abstract

How complicated is the relationship between a protein’s sequence and its function? High-order epistatic interactions among residues are thought to be pervasive, making a protein’s function difficult to predict or understand from its sequence. Most prior studies, however, used methods that misinterpret measurement errors, small local idiosyncracies around a designated wild-type sequence, and global nonlinearity in the sequence-function relationship as rampant high-order interactions. Here we present a simple new method to jointly estimate global nonlinearity and specific epistatic interactions across a protein’s genotype-phenotype map. Our reference-free approach calculates the effect of each amino acid state or combination by averaging over all genotypes that contain it relative to the global average. We show that this method is more accurate than any alternative approach and is robust to measurement error and partial sampling. We reanalyze 20 combinatorial mutagenesis experiments and find that main and pairwise effects, together with a simple form of global nonlinearity, account for a median of 96% of total variance in the measured phenotype (and > 92% in every case), and only a tiny fraction of genotypes are strongly affected by epistasis at third or higher orders. The genetic architecture is also sparse: the number of model terms required to explain the vast majority of phenotypic variance is smaller than the number of genotypes by many orders of magnitude. The sequence-function relationship in most proteins is therefore far simpler than previously thought, and new, more tractable experimental approaches, combined with reference-free analysis, may be sufficient to explain it in most cases.

If we had a comprehensive understanding of a protein’s sequence-function relationship, we could predict the functional and evolutionary consequences of any mutation or novel amino acid sequence. Whether such knowledge is possible in practice depends on the extent of epistatic interactions. If all residues in a protein act independently, then knowing the effects of point mutations on any genetic background would suffice to predict the function of any possible sequence, and any mutation’s evolutionary fate would be independent of the genetic context in which it occurs. A simple genetic architecture like this could be easily inferred using moderate-throughput experiments. At the opposite extreme, extensive high-order epistasis would cause each mutation to have idiosyncratic effects that depend absolutely on the particular sequence background into which it is introduced. In that case, assessing the protein’s genetic architecture would require exhaustive assessment of every possible genotype, and the evolutionary accessibility of all mutations would change with every sequence substitution that occurs.

Deep mutational scanning (DMS) methods to characterize large libraries of protein variants have recently made it possible to assess the complexity of the sequence-function relationship, but studies to date disagree on the complexity of the sequence-function relationship. Some report extensive high-order interactions (1–7), while others find that they account for less than 10% of functional variance among sequences (8–16). Even pairwise interactions are pervasive and strong in some studies (7, 12, 17–21) but sparse and weak in others (9, 16, 22). In terms of overall complexity, some report a sparse genetic architecture in which only a small fraction of possible terms are important (13, 13, 16, 16) but others point to a much more complex mapping in which many different states and combinations shape the sequence-function relationship (7, 18, 20, 21).

These discrepancies may reflect the use of different methods to characterize epistasis. Two aspects of widely used approaches can yield overestimates of amino acid interactions. First, most studies to date have analyzed mutational data using reference-based models, which designate a single sequence as the reference against which all effects are measured: the main effects of mutations are estimated by introducing each one into a single reference genotype, and epistatic interactions are calculated as the deviation of a protein variant containing several mutations from the sum of the lower order effects. A concern is that technical noise or small epistatic idiosyncracies in measurement of the reference genotype or low-order variants can propagate into estimates of higher-order effect terms, causing spurious higher-order interactions to be inferred (23). Second, many studies have not fully accounted for nonspecific epistasis, which arises from a global nonlinear relationship between sequence and phenotype that affects all mutations identically, such as diminishing fitness returns or the relationship between protein stability and protein function (24–27). If this nonlinearity is not adequately addressed, spurious specific interactions must be invoked to explain why every mutation’s effects differ among genetic backgrounds.

We therefore developed a method that does not suffer from these sources of error and used it to systematically reexamine existing datasets. Advances have been made in both potential areas of concern, but currently available methods still have critical limitations. Fourier analysis (28, 29)—also known as simplex encoding (30) or graph Fourier transform (31)—is reference-free: it averages the effects of sequence states across many genetic backgrounds and defines them relative to the global average over all genotypes, and is therefore likely to improve robustness to measurement error and local idiosyncrasies. This approach can be implemented as simple linear regression when sampling is limited to just two amino acid states per site (32). For datasets with more than two states, however, current implementations require complex matrix algebra, such as building and manipulating large Hadamard matrices or constructing graph Fourier bases, and the resulting model terms are intelligible only with respect to these matrices. Because of this complexity, only one multi-amino acid dataset has been analyzed using this approach (31). A third formalism-background-averaging (BA) (23), also known as the Walsh-Hadamard transform (2, 33)—has also been developed. This approach, which has been applied only to two-amino acid datasets (but see ref. (34) for an application to tRNA), occupies a middle ground between reference-based and Fourier analyses: it averages mutational effects over backgrounds, but it defines them relative to a particular reference state at each site rather than to a single reference genotype.

Existing methods to address nonspecific epistasis also have limitations. Sometimes molecular phenotypes can be measured or transformed onto a scale that is not strongly affected by nonspecific epistasis, such as free energy of binding within the dynamic range of assay measurement (16, 35, 36). But many phenotypes scale nonadditively because of multiple and complex causes, and the appropriate transformation to account for nonspecific epistasis can therefore seldom be known in advance (37). Several studies have addressed this problem by estimating from the data a transformation that minimizes nonadditivity in the relationship between the measured phenotype and the estimated main effects of mutations (9, 11, 13, 22, 38–40). Many of these studies use rigid convex or concave transformations that cannot incorporate the most important kinds of nonlinearity, such as the bounding of phenotypic measurements within upper and lower limits, a pattern that has been observed in many DMS studies (9, 22, 38); bounding can occur if measurement assays have limited dynamic range and/or the biochemical processes that produce molecular phenotypes have an intrinsic floor and/or ceiling, such as that produced by the relationship between the free energy of folding/binding and the probability that a protein occupies a functional state. Some studies have used a flexible spline model or neural network (9, 22, 38) to model nonspecific epistasis, but these methods have not been widely adopted because they are cumbersome to implement and difficult to interpret.

Here we develop and implement a straightforward formulation of reference-free analysis that is applicable to any number of states, and we couple it in a joint estimation procedure with an effective model of nonspecific epistasis. We first explain our approach and compare its desirable properties to existing approaches. We then use it to reanalyze 20 previously published combinatorial mutagenesis experiments on proteins with diverse functions, and we use the results to assess the complexity of the sequence-function relationship. Finally, we explore strategies to infer sequence-function relationships when only a fraction of possible genotypes can be experimentally sampled.

## Results

### Reference-free analysis of genetic architecture.

Our method of reference-free analysis defines the causal factors in a protein’s genetic architecture as sequence states rather than mutations. This structure allows it to describe the genetic causes of phenotypic variation across the ensemble of all genotypes. In reference-based and background-averaged analyses, the determinants of genetic architecture are mutations—changes from the reference state to a different state—rather than the states themselves. Proteins containing a reference state therefore have no genetic determinant for that state at any site or for any combination across sites that includes even one reference state. For example, the “wildtype” sequence contains the reference state at all sites: it has no mutations, so it manifests no main effects or epistatic interactions at all. All the single-step neighbors of the reference are each subject to one main effect, but they contain no combinations of mutations, so they cannot be affected by epistasis at any order. Two-step mutants are subject to one pairwise epistatic effect each but cannot be affected by higher-order epistasis, and so on. In fact, all these “low-order” genotypes are proteins too, and their genetic architecture is just as interesting and complex as protein sequences distant from the wild-type.

**Fig. 1.**
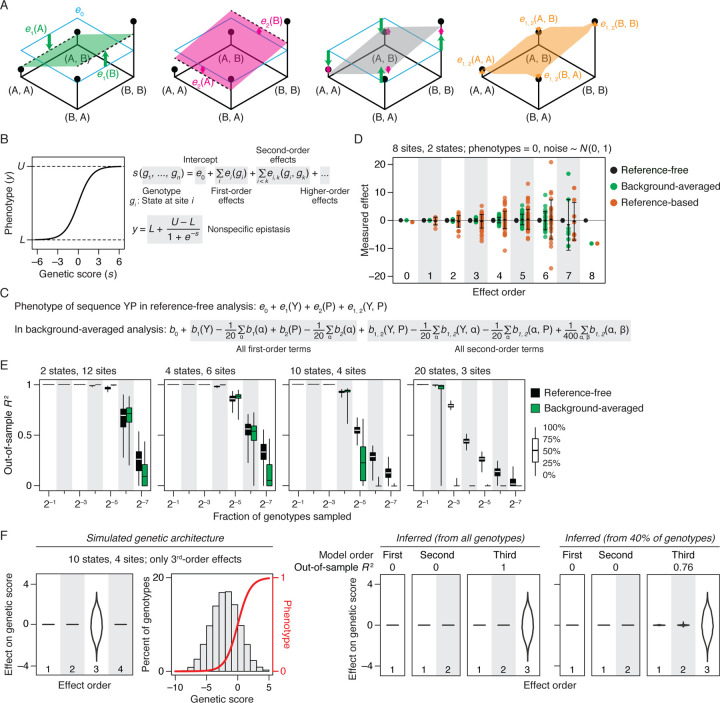
Reference-free analysis (RFA) of genetic architecture. (*A*) Illustration of RFA on a two-site, two-state genotype space. The four possible genotypes (dots) are arranged on a plane with the phenotype of each indicated by elevation. The zero-order term (*e*_0_) is the average phenotype of all genotypes, marked by the horizontal cyan plane. (*Left panel* ) The first-order effect of state A or B at site 1 [*e*_1_(*A*) or *e*_1_(*B*), green arrows] measures how the average phenotype of all genotypes containing that state (dashed line) differs from the global average; these terms in the model are represented by the green plane, which predict the phenotype of any genotype based on its state at site 1. (*Second panel* ) First-order effects at site 2 [*e*_2_(*A*) and *e*_2_(*B*)] are defined similarly and represented with pink arrows and plane. (*Third panel* ) The complete first-order model predicts the phenotype of each genotype as the sum of the first-order effects of all its sequence states plus the global average, represented as the grey plane tilted in both dimensions. (*Right panel* ) The pairwise interaction between states A and B at sites 1 and 2 [*e*_1,2_(*A, B*), orange] measures how the average phenotype of all genotypes containing the two states [here just one genotype (A, B)] differs from the first-order prediction. (*B*) We implement RFA with a sigmoid link function to incorporate nonspecific epistasis. Each variant’s genetic score (*s*) is the sum of the effect of each state and state-combination it contains. A sigmoid link function transforms *s* of each variant into its phenotype, *y*. Parameters *L* and *U* represent the lower and upper bound of measurable phenotype. (*C*) Mapping of genetic effects to phenotype with 20 possible states per site using RFA and background-averaged analysis, shown for an example two-site variant containing states Y and P. *e*, RFA genetic effects as defined in panel (*A*); *b*, background-averaged genetic effects for each possible amino acid state (*α*) and pair (*α, β*). (*D*) RFA is robust to measurement noise. For an eight-site, two-state genotype space, phenotype data were simulated under a genetic architecture in which all true phenotypes and genetic effects equal zero but measurement is subject to Gaussian noise of variance 1. Effects were then estimated using reference-free, background-averaged, and reference-based formalisms. Each dot is an estimated effect at the specified order. Error bars, standard deviation. (*E* ) RFA is more robust to missing genotypes than is background-averaged analysis. Phenotypes were simulated across four genotype spaces with different numbers of states per site under a genetic architecture in which first- and second-order effects account for 40 and 60% of phenotypic variance, respectively. After removing an increasing fraction of genotypes, a second-order RFA or background-averaged model was inferred and used to predict the phenotype for the removed genotypes. Boxplot, distribution of out-of-sample *R*^2^ across 200 trials; negative *R*^2^ values are plotted as zero. (*F* ) RFA does not misinterpret high-order epistasis as clusters of lower-order interactions. Phenotypes were simulated on a four-site, ten-state genotype space with only third-order determinants (distribution shown in the first panel) and a sigmoid relationship between genetic score and phenotype (second panel). (*Right panels*) RFA models of first, second, and third-order were fit to these data. The distribution of inferred effects and the fraction of variance explained are shown for when the models are fit to all genotypes or a random subset.

Reference-free analysis (RFA) allows all genotypes to provide equally important evidence about the global genetic architecture. RFA takes an ANOVA-like approach in which every sequence state at every site is a causal factor that can potentially affect the functional phenotype, and all such factors can interact with each other. A combinatorial DMS study represents a full factorial experiment from which all possible causal factors and all possible interactions can be quantified (Fig. 1A). In the absence of nonspecific epistasis, the model is structured so that each protein’s phenotype is the simple sum of the functional effects of all its states and combinations. The model’s zero-order term, which affects all sequences, is the average phenotype across all genotypes. The first-order terms are the main effects of each amino acid state at every variable site in the sequence, which are defined as the difference between the average phenotype of all variants containing a state of interest and the global average. The interaction terms at each increasing order are the epistatic effects of every pair, triplet, or higher-order combination, defined as the difference between the average phenotype of all variants containing that set of states and the expected deviation from the global average given the relevant lower-order effects.

To incorporate nonspecific epistasis, we use a generalized linear model in which each protein’s phenotype is a nonlinear function of its genetic score—the sum of the specific effects of the states and their combinations in the protein’s sequence (Fig. 1B). To incorporate phenotypic bounding, we use a sigmoid link function, which contains only two parameters—the maximum and minimum observable phenotype—to transform genetic score into phenotype.

RFA has several desirable features. Setting aside the link function for simplicity of explanation, the RFA model at each order explains the maximum amount of phenotypic variance across all measured genotypes that could possibly be explained by any linear model of the same order (SI Appendix). Consider the zero-order RFA model, in which the only term is the mean phenotype across all genotypes; this estimator minimizes the mean squared error between measurement and prediction across all variants and therefore is the best possible single-parameter predictor (Fig. 1A). In the first-order RFA model, the predicted phenotype of a variant is the sum of all the main effects of its constituent amino acids plus the global average; because each main effect is calculated as the deviation of the average phenotype of all variants containing some amino acid state from the global average, this set of predictors again minimizes the mean squared error across all variants and maximizes the phenotypic variance explained compared with any other first-order model (SI Appendix). This model structure and its desirable properties extend to each increasing order.

Reference-free analysis contrasts with reference-based analysis (RBA), which defines each effect in the model using single measurements rather than averages. The RBA zero-order term is the phenotype of the designated reference sequence; this estimator is a good predictor in the local neighborhood of the reference but is less accurate across sequence space than the global average. The first-order RBA term for each state is the difference between the one mutant that contains that state and the reference sequence, and each higher-order term is the difference between the one mutant containing a combination of states and the sum of the estimated lower-order effects. These are good predictors of the effects of introducing each state or combination into the reference background, but they are suboptimal estimators across the set of all genotypes. RFA also differs from background-averaged analysis (BA), which designates a particular state as the reference at each site; the main effect of each amino acid is defined as the average difference in phenotype of the set of variants containing that state and the set of variants containing the reference state at the same site.

The structure of RFA has several additional advantages. First, the mapping from reference-free effects to phenotype is intuitive. Each variant’s genetic score is a simple sum of the effects of its sequence states and combinations. This contrasts with BA and prior implementations of Fourier analysis, where the genetic score of each variant is a complicated weighted sum of every term in the entire model, including the terms for states and combinations that the variant does not contain (Fig. 1C). Second, RFA facilitates direct quantification of the portion of all phenotypic variation that is caused by any term or set of terms in the model using a simple ANOVA-like framework. Because RFA terms are defined as mean deviations from the global average, they have a straightforward relationship to variance: The variance attributable to any RFA term is the square of its magnitude normalized by the fraction of all variants that contain the state or combination. The contribution of any set of terms—such as all terms at some particular order or some set of sites—is the sum of the individual contributions (SI Appendix).

### Robustness to measurement noise and partial sampling.

If we had precise phenotypic measurement for every possible variant, we could exactly compute the effects of genetic states and combinations as they are encoded in any of the formalisms. In reality, experimental data are always affected by measurement noise, and in large libraries some variants typically go unmeasured. RFA is designed to perform well in the face of both these challenges.

To assess the performance of RFA versus RBA and BA when measurements are noisy, we simulated phenotypic measurements using a known genetic architecture and normally distributed measurement error. We then estimated the genetic architecture from these data and compared the estimated model terms to the true values under each approach (Fig. 1D). We found that RFA yields estimated effect terms that are precise and unbiased. By contrast, the average error in RBA’s model terms is 50 times greater than in RFA, and the error increases systematically with epistatic order. For background averaging, the error in first-order terms is about twice that of RFA, but errors grow quickly as the order of epistasis increases, reaching a maximum at high orders that is 100-fold worse than RFA.

When data are incomplete, the model terms of RFA and BA can still be estimated using regression because each term is averaged over many particular genotypes, and the phenotypes of unmeasured variants can then be predicted from the estimated model. In both cases, terms estimated by regression should converge to the true effects as sample size increases, and the estimates are unbiased when variants are sampled without bias (SI Appendix). (Regression cannot be used with RBA, because any missing variant makes it impossible to estimate the model term signified by that variant and all terms above that order that depend on it.)

To characterize the relative power and accuracy of regression-based RFA and BA with incomplete data, we simulated data using a simple genetic architecture, removed a variable fraction of variants from the dataset, fit the models to the remaining data by regression, and used the best-fit model to predict the phenotypes of the excluded variants (Fig. 1E). We found that when there are only two or four states per site, both RFA and BA have high predictive accuracy, with a decline only after the fraction of sampled genotypes drops to 0.1%, at which point RFA is slightly more accurate. When there are 10 or 20 possible states, however, RFA predictions were much more accurate and robust than BA, the accuracy of which degraded rapidly as the sample size shrank. With 20 states per site, BA became completely uninformative when sample density dropped below 25%, whereas RFA maintained some predictive value even at much lower sampling densities. The structure of the formalisms explains RFA’s superior performance in the face of measurement noise and partial sampling. In RFA, every measurement in the dataset is used to calculate each model term. Averaging over so many measurements dramatically reduces the influence of individual errors: the expected error in RFA terms is always smaller than that of individual phenotypic measurements, is negligible for low- and medium-order terms, and increases slowly with epistatic order. By contrast, RBA calculates each term as the difference between individual variants, without any averaging; epistasis must be invoked whenever the phenotype of a variant deviates from the sum of its lower order effects, which themselves were calculated from the deviation of single genotypes from the reference. Because each RBA term is a chain of sums and differences of many individual measurements, error variance propagates: the expected error in any RBA term is always greater than that of individual measurements and it snowballs with order, so in practice high- and even medium-order terms cannot be estimated with reasonable accuracy. For the same reason, if there are small local idiosyncracies in the phenotype of the wild-type or low-order mutants caused by higher-order epistasis, these deviations will propagate into increasingly large estimates of high-order interactions as distance from the reference grows. By estimating each effect as an average across numerous genetic backgrounds, BA reduces error propagation compared to reference-based analysis. But differences are still defined relative to a particular reference state rather than the global average, so the number of genetic backgrounds for averaging is smaller than in RFA and the sensitivity to measurement noise in each term is therefore greater. The number of relevant genetic backgrounds for estimating each BA term declines exponentially with the epistatic order, so the expected error in those terms also increases exponentially, becoming as large as the error of RBA at the highest orders. Moreover, BA predicts the phenotype of an unsampled variant as a weighted sum of every single term in the model, whereas RFA uses only the terms for the states and combinations in the variant’s sequence (Fig. 1C). Errors in estimated model terms caused by noise therefore propagate in BA’s phenotype predictions, and this effect is exacerbated as more states per site are considered, because the total number of terms in the model increases exponentially with the number of states. RFA is insensitive to the number of states, because it predicts a variant’s phenotype using only the terms for the states that are contained in its sequence. Alternative implementations of Fourier analysis are structured similarly to BA in mapping the terms to phenotype (SI Appendix), so they are expected to be more sensitive to noise and partial sampling than RFA.

### Reference-free analysis does not oversimplify genetic architecture.

We explored the possibility that RFA might oversimplify genetic architecture by misinterpreting high-order interactions as clusters of lower-order effects. The model is structured so that each order of reference-free effects produces a distinct pattern of phenotypic variation, and the pattern produced by effects at one order cannot be explained by model terms at another (SI Appendix). High-order variation appears as noise around the mean at lower orders, so a truncated low-order RFA model cannot explain any phenotypic variation caused by unmodeled higher-order interactions. The complexity of genetic architecture can therefore be accurately gauged by fitting truncated models and determining how much phenotypic variance is explained (SI Appendix).

To verify that RFA in practice does not oversimplify genetic architecture—particularly when nonspecific epistasis is present and sampling is incomplete—we used simulations in which phenotypes are generated by a genetic architecture that contains only third-order effects plus nonspecific epistasis. We then fit RFA models truncated at various orders to these data (Fig. 1F). First- and second-order truncated models correctly explain zero phenotypic variance and detect no first- or second-order effects. When the third-order model is used, all variance is correctly attributed to third-order interactions. Similar results obtain when variants are only partially sampled.

### Simplicity of protein sequence-function relationships.

To understand the genetic architecture of real proteins, we used RFA to analyze 20 published experiments that characterized mutant libraries in a variety of protein families with different types of functions: antibodies, enzymes, fluorescent proteins, transcription factors, viral surface proteins, and toxin-antitoxin systems. We considered only datasets in which combinatorial libraries were used and measurements had high reproducibility (*r*^2^ > 0.9 among replicates; Table 1). We focused primarily on deep mutational scans of large libraries, but we included three small datasets in which high-order epistasis has been reported. The datasets range in size from 32 to 160,000 possible genotypes, with the number of variable sites ranging from 3 to 16 and the number of states per site from 2 to 20.

**Fig. 2.**
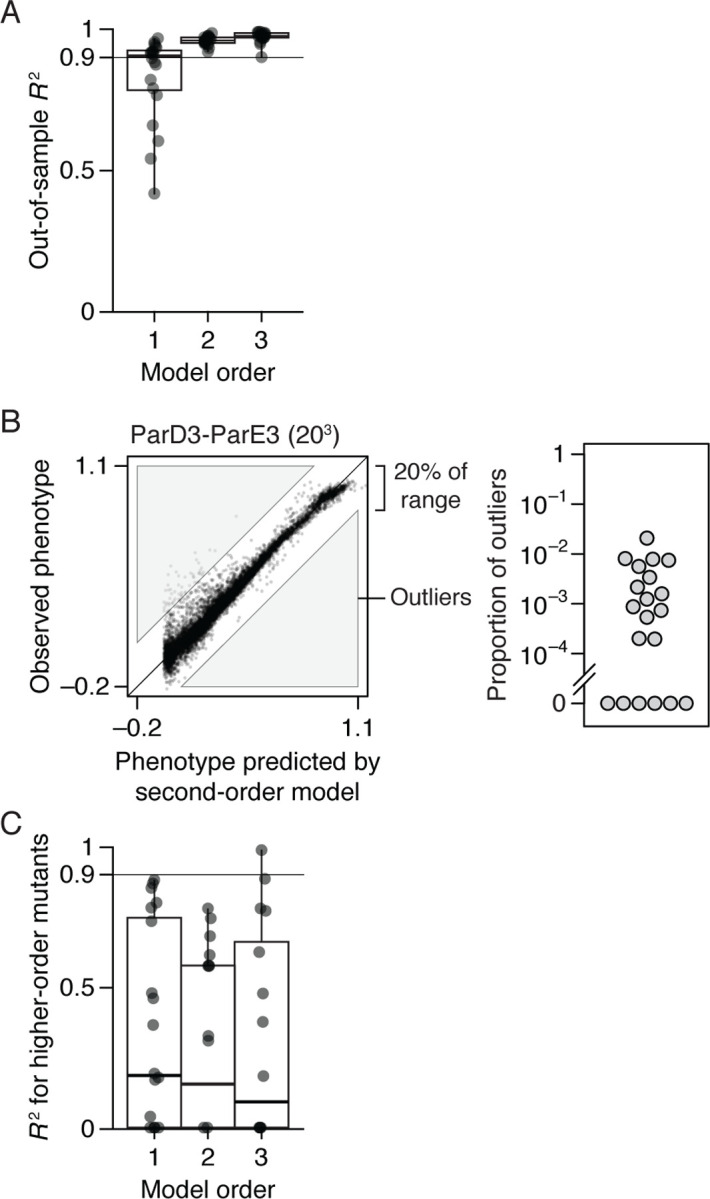
Simplicity of protein sequence-function relationships. (*A*) RFA of 20 combinatorial mutagenesis datasets (Table 1). First-, second-, and third-order models with the sigmoid link function were evaluated by cross-validation—by inferring the model from a subset of data and predicting the rest of data. Each dot shows the mean out-of-sample *R*^2^ for one dataset; boxplots show the median, interquartile range, and total range across datasets. SI Appendix, Fig. S1, shows the *R*^2^ for individual datasets. (*B*) Variants possibly affected by strong high-order epistasis were identified as outliers in the second-order model (residual greater than 20% of the phenotype range). (*Left* ) Outliers in the ParD3-ParE3 (20^3^) dataset. Each point is a variant, plotted by its observed and predicted phenotype. (*Right* ) Proportion of outliers in each dataset. (*C*) Reference-based analysis of the 20 datasets. Each model was evaluated by predicting the phenotypes of higher-order mutants. Nonspecific epistasis was accounted for as in (*A*), and the wild-type genotype was used as reference. Negative *R*^2^ values are plotted as zero.

We first assessed the extent to which main effects alone explain the genetic architecture by fitting a truncated first-order reference-free model, with the sigmoid link function to incorporate nonspecific epistasis. Using cross-validation to estimate the fraction of phenotypic variance explained, we found that the first-order model achieves a median out-of-sample *R*^2^ of 0.91 across all 20 datasets, a maximum of 0.97, and > 0.75 in all but four cases (Fig. 2A). There is no clear relationship between the amount of variance explained by main effects and the number of sites or states assayed (SI Appendix, Fig. S1): the 11 datasets with *R*^2^ > 0.9 include two-state, 16-site experiments in which up to 16^*th*^-order epistasis is theoretically possible (CR9114-B and H3) and a four-site, 20-state experiment in which the 80 main effects account for 92% of phenotypic variance (ParB). The additive effects of individual amino acids therefore account for the majority of genetic variation in protein function in most cases.

When second-order terms are included, virtually all genetic variance is explained, with a median cross-validation *R*^2^ of 0.96 and a minimum of 0.92 across all datasets (Fig. 2A). Adding third-order terms offers only marginal or no improvement in fit (median change in out-of-sample *R*^2^ of 0.02, maximum 0.04). The small fraction of phenotypic variance unexplained by the third-order model represents some combination of fourth- and higher-order epistasis, measurement noise, and limitations in the sigmoid link function to accurately capture nonspecific epistasis.

Although high-order epistasis is negligible for the majority of genotypes, there could still be a subset of genotypes shaped by strong high-order epistasis. To address this possibility, we analyzed the residuals of the second-order model, which represent the sum of all higher-order epistatic interactions and measurement noise. Genotypes with a residual greater than 20% of the phenotype range were considered candidates for strong higher-order epistasis (Fig. 2B), although erratic measurement noise cannot be excluded. The proportion of such genotypes is zero in six datasets and between 0.02% and 2% in the others. Strong high-order epistasis therefore affects a tiny fraction of genotypes.

These data establish that protein sequence-function relationships are surprisingly simple: estimating just additive effects and pairwise interactions, coupled with a simple model of nonspecific epistasis, is sufficient for high-accuracy phenotypic prediction across the entire ensemble of protein variants. Third- and higher-order interactions are not completely absent, but these effects are typically weak, and each one affects a small number of genotypes.

Finally, we asked whether using RBA instead of RFA would produce spurious inference of epistasis from these datasets. We fit first-, second-, and third-order RBA models (including the sigmoid link function) to a designated wild-type and all single, double, and triple mutants; the phenotypes of other genotypes were then predicted using the best-fit model parameters, and the *R*^2^ was calculated. We found that RBA’s accuracy is dramatically lower than RFA’s: The median *R*^2^ across datasets is less than 0.2 at all orders, leaving the vast majority of genetic variance to be explained by higher-order epistasis (Fig. 2C). The fraction of variance attributable to each epistatic order fluctuates dramatically with the protein chosen as the reference (SI Appendix, Fig. S2). Using the published “wild-type” sequence does not systematically attribute less or more variation to epistatic orders compared with using random reference sequences.

### The primary cause of nonspecific epistasis is phenotype bounding.

**Fig. 3.**
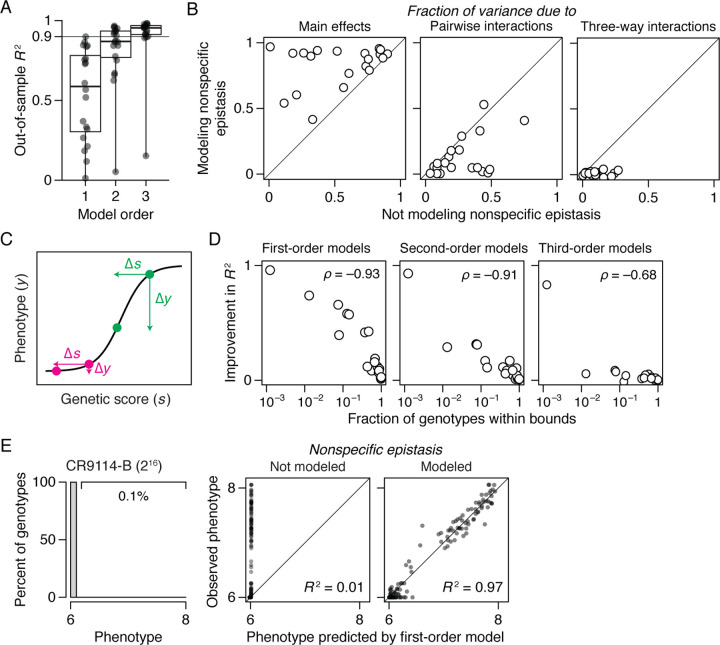
The primary cause of nonspecific epistasis is phenotype bounding. (*A*) RFA of the 20 datasets without incorporating nonspecific epistasis, shown as in Fig. 2A. (*B*) Incorporating nonspecific epistasis reduces the amount of phenotypic variance attributable to pairwise and higher-order interactions. Each dot shows the variance component for one dataset computed with or without incorporating nonspecific epistasis. (*C*) Nonspecific epistasis causes the phenotypic effect of a mutation (∆*y*) to vary among genetic backgrounds (magenta versus green) even when the effect on genetic score (∆*s*) is constant. Phenotype bounding is a particularly strong form of nonspecific epistasis that causes mutations to appear neutral on backgrounds near the bounds but not on others. (*D*) The extent to which the sigmoid link function improves the model fit (comparing out-of-sample *R*^2^ in Fig. 3A versus 2*A*) is proportional to the fraction of genotypes at or beyond the phenotype bounds. (*E* ) In a dataset where only 0.1% of genotypes are within the bounds, incorporating nonspecific epistasis raises the fraction of phenotypic variance attributable to main effects from 0.01 to 0.97.

We next characterized the effect of incorporating nonspecific epistasis in the 20 datasets by comparing the results of RFA with and without the sigmoid link function. We found that incorporating nonspecific epistasis dramatically improves phenotype prediction, increases the variance attributable to main and low-order epistatic effects, and reduces that attributed to high-order specific epistasis (Fig. 3, *A* and *B*). For the first-order reference-free models, using the link function improves the median out-of-sample *R*^2^ from 0.59 to 0.92. With second-order models, the sigmoid link function improves the median *R*^2^ from 0.87 to 0.96. With third-order models, median *R*^2^ improves from 0.95 to 0.98.

The dramatic improvement in fit conferred by the simple sigmoid function suggests that phenotype bounds—biological or technical limits on the dynamic range over which genetic states have measurable effects on function—are the primary cause of nonspecific epistasis in most proteins (Fig. 3C). Corroborating this conclusion, the degree of improvement in *R*^2^ when the sigmoid link function is used is tightly correlated with the fraction of genotypes at or beyond the phenotype bounds (Fig. 3D). For example, in the CR9114-B dataset, 99.9% of genotypes are at the lower bound and the out-of-sample *R*^2^ of the first-order model rises from 0.01 to 0.97 by incorporating nonspecific epistasis (Fig. 3E). By contrast, modeling nonspecific epistasis has a modest impact when most genotypes lie within the dynamic range.

Taken together, these findings indicate that limited range of measurable phenotypic variation is the primary cause of nonspecific epistasis in deep mutational scanning datasets, and that incorporating it using a simple link function can yield a dramatic improvement in fit and reduce spurious inferences of specific epistasis, including at high orders. Although the mechanisms underlying global nonlinearity in the genotype-phenotype relationship are likely to be complex and to vary among proteins, the simple sigmoid link function effectively captures its most salient features.

### Sparsity of protein sequence-function relationships.

**Fig. 4.**
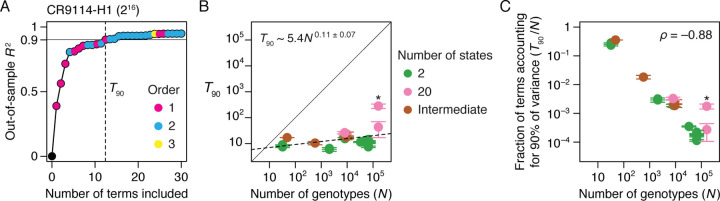
Sparsity of protein sequence-function relationships. (*A*) Measuring the sparsity of genetic architecture illustrated on the CR9114-H1 dataset. Reference-free effects were estimated using a third-order model and then ranked by the fraction of variance they explain. Models of increasing complexity were then constructed by sequentially including each effect term, and each model was evaluated by cross-validation. Each dot represents a model, colored by the order of the last term added. Vertical line marks *T*_90_, the minimal number of terms required for an out-of-sample *R*^2^ of 0.9. (*B*) *T*_90_ as a function of the total number of genotypes. Dotted line, best-fit power function. Asterisk, GB1 dataset. Each *T*_90_ was estimated in two ways: as the number of terms required to reach *R*^2^ of 0.9 (upper error bar)—an overestimate because measurement noise prevents any model from attaining out-of-sample *R*^2^ of 1—and as the number of terms required for an *R*^2^ equal to 90% of that of the complete third-order model (lower error bar). Circles show the average of the two estimates. (*C*) Fraction of all possible reference-free terms that account for 90% of phenotypic variance plotted versus the total number of genotypes. Asterisk, GB1 dataset.

Next, we asked whether protein function across the 20 datasets tends to be dictated by a few large-effect amino acid states/combinations or by many determinants of small effects. To quantify the sparsity of each protein’s genetic architecture, we estimated the minimal number of model terms required to predict the function with 90% accuracy (*T*_90_). We calculated each protein’s *T*_90_ by ranking all the effects in the protein’s genetic architecture by their contribution to phenotypic variance, constructing increasingly complex RFA models by sequentially including each effect term, and estimating the predictive accuracy of each model using cross-validation (Fig. 4A).

We found that genetic architecture is very sparse (Fig. 4B). *T*_90_ ranges from just 6 to 44 terms across all datasets except for the GB1 dataset (282 terms), in which the mutated sites were specifically chosen to be enriched for epistatic interactions (10). *T*_90_ increases very slowly with the size of genotype space, so the fraction of all possible terms that must be included to reach *R*^2^ of 0.90 (*F*90) declines approximately linearly as the number of possible genotypes rises (Fig. 4C). This relationship holds irrespective of the number of states per variable site. Taken together, our findings suggest that even very large genetic architectures should be describable with a compact set of important terms. For example, for a genotype space of two states at 100 variable sites ( 10^30^ genotypes and the same number of possible model terms), the expected *T*_90_ is less than 10,000 terms.

### Estimating genetic architecture by random sampling.

Even though only a small fraction of terms is important in proteins’ genetic architecture, finding them may be challenging. Experimentally analyzing exhaustive libraries is intractable for more than a small number of sites. A critical question is therefore whether genetic architecture can be estimated by a sparse learning approach that characterizes a relatively small random sample of possible genotypes and uses penalized regression to estimate from these data the most important effects in the genetic model (13).

**Fig. 5.**
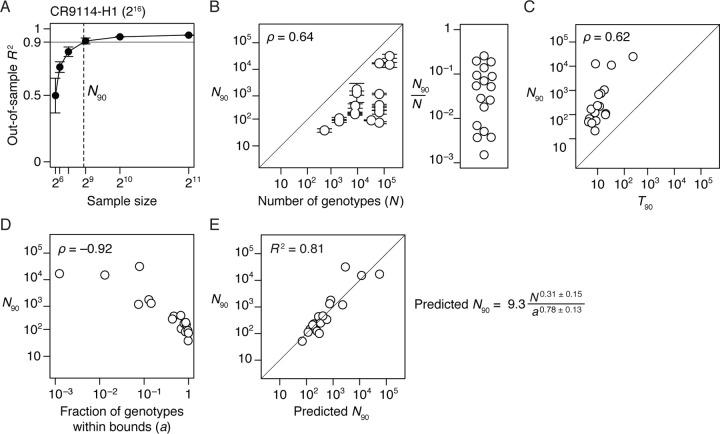
Learning the genetic architecture by random sampling. (*A*) Learning by random sampling illustrated on the CR9114-H1 dataset. Up to third-order reference-free effects were inferred from a varying number of randomly sampled genotypes and were evaluated by predicting the phenotypes of all unsampled genotypes. For each sample size, mean and standard deviation of out-of-sample *R*^2^ across 10 trials are shown. Dashed line marks *N*_90_, the minimal sample size required for mean out-of-sample *R*^2^ of 0.9. (*B*) (*Left* ) *N*_90_ as a function of the total number of genotypes (*N* ). Error bars were computed as in Fig. 4B. The three datasets with 48 or fewer genotypes are not shown. (*Right* ) Distribution of the fraction of genotypes that must be sampled to account for 90% of phenotypic variance. (*C*) *N*_90_ as a function of *T*_90_, the minimal number of reference-free effects required to explain 90% of phenotypic variance (Fig. 4). (*D*) *N*_90_ as a function of the fraction of genotypes within phenotype bounds. (*E* ) Modeling *N*_90_ as a power function of the total number of genotypes (*N* ) and the fraction of genotypes within phenotype bounds (*a*). The best-fit curve is shown along with standard errors.

To characterize the fraction of genotypes that must be sampled to reconstruct the genetic architecture of each dataset, we simulated sparse learning by randomly sampling a variable number of genotypes and using penalized regression to estimate the RFA terms. We then predicted the phenotypes of the unsampled genotypes, calculated the out-of sample *R*^2^, and determined the minimum sample size required for *R*^2^ of 0.9 (*N*_90_; Fig. 5A).

We found that genetic architecture cannot be reliably estimated by sparse random sampling. Excluding the three small datasets, *N*_90_ ranges from 0.2 to 25% of the total number of genotypes, with a median of 5% (Fig. 5B). Even the lowest end of this range does not bode well for estimating genetic architecture in large sequence spaces that contain billions or more genotypes.

We explored several factors that might determine the required sampling density: the total number of genotypes in the sequence space, the sparseness of the architecture, and the fraction of genotypes with phenotypes in the dynamic range of measurement. First, the genetic model for a larger sequence space entails more potential terms at every epistatic order, so estimating it might require sampling a larger library. We found that *N*_90_ does increase with the number of total possible genotypes, but there is considerable scatter in this relationship (Fig. 5B). Second, one might expect that estimating a simple genetic architecture requires a smaller sample than a more complex architecture. We found a weak relationship between the number of model terms required to explain 90% of the phenotypic variance (*T*_90_) and the number of genotypes that must be sampled to achieve this level of explanation (*N*_90_) (Fig. 5C). An extreme case is the CR9114-B dataset (total 2^16^ = 65,536 genotypes), in which just ten main effects explain 90% of the variance but 16,000 genotypes—about 25% of the total—must be sampled to find them.

Finally, we considered whether the masking of phenotype by the upper or lower bound might be a factor in the effectiveness of sampling strategies. Genotypes with phenotypes at or near these limits contribute little quantitative information about the effects of the states they contain, so if most variants in a library are at the bounds, then very large samples might be required to obtain information about the genetic architecture. We found a strong negative relationship between *N*_90_ and the fraction of variants in the dynamic range (Fig. 5D). In the CR9114-B dataset discussed above, for example, 99.9% of all variants are at the lower bound, so the 16,000 variants required to reach *N*_90_ only contain about 16 genotypes in the dynamic range. Conversely, in the CH65-MA90 dataset, there are > 65, 000 total genotypes, but the architecture can be estimated from a sample of just 99 variants because virtually all of the data are within the dynamic range.

The size of sequence space (*N* ) and the fraction of variants in dynamic range (*a*) are therefore the key factors that determine how well a genetic architecture can be reconstructed by random sampling. To quantify the effects of these factors, we modeled *N*_90_ as a function of *N* and *a* across the datasets (Fig. 5E). The inferred relationship allows us to predict how large a sample should be required to estimate a genetic architecture given the size of the sequence space and the fraction of variants in dynamic range. If all genotypes in the CR9114-B dataset were in the dynamic range, a sample of only 300 variants would need to be measured, rather than the 16,000 actually required. But some sequence spaces are so large that estimating their genetic architecture by random sampling would not be practical, even if dynamic range were unlimited: for the two-state, 100-variable-site protein described above, it would still be necessary to measure 20 billion variants, even though only ~ 10, 000 terms are expected to account for 90% of phenotypic variance.

We conclude that despite the simplicity of proteins’ genetic architecture, its most important causal factors cannot be efficiently estimated by random sampling using experimental libraries, in which the majority of variants are typically nonfunctional. It is therefore important to develop an efficient non-random sampling strategy to identify the important main and pairwise effects in a protein’s genetic architecture. Characterizing libraries of low-order combinations in diverse functional homologs, rather than attempting complete combinatorial scans in a single protein, may be effective. Improvements that expand the dynamic range of deep mutational scan experiments will also help.

### Understanding genetic architecture.

**Fig. 6.**
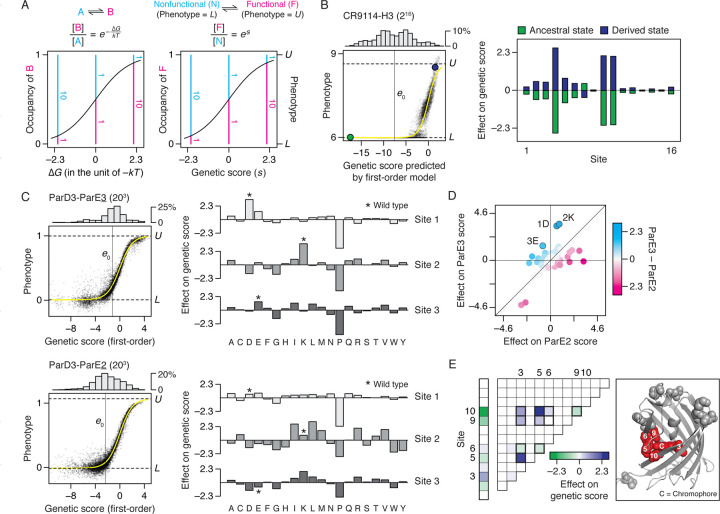
Understanding genetic architecture. (*A*) Interpreting genetic score (*s*) as Gibbs free energy difference (∆*G*). (*Left* ) Relative occupancy of two thermodynamic states as a function of their ∆*G*. *k*, Boltzmann constant; *T*, absolute temperature. (*Right* ) Our sigmoid model of nonspecific epistasis corresponds to an equilibrium between two states—the “functional” state, of phenotype of *U*, and the “nonfunctional” state, of phenotype of *L*. Their relative occupancy (pink and blue lines) equals *e*^*s*^, allowing *s* to be interpreted as *−*∆*G* in the unit of *kT*. (*B*) Analysis of the CR9114-H3 dataset, which measures the affinity of all possible combinations of ancestral and derived amino acids at 16 sites in an antibody towards an influenza hemagglutinin. (*Left* ) First-order RFA. Each dot is a genotype, plotted by its measured phenotype and estimated genetic score. Histogram, distribution of genetic score; yellow curve, inferred nonspecific epistasis; horizontal lines, phenotype bounds; vertical line, global average; green and purple dots, ancestral and derived genotypes. (*Right* ) First-order effects of amino acids at each site. (*C*) ParD3-ParE3 and ParD3-ParE2 (20^3^) datasets, which measure how all possible variants of the protein ParD3 at three sites bind to ParE3, the cognate substrate, or ParE2, a noncognate substrate. (*Left* ) First-order RFA shown as in (*B*). (*Right* ) First-order effects of amino acids at each site. Asterisk, wild type. (*D*) Comparing the effect of each amino acid on ParE3 versus ParE2 binding. Wild type amino acids are marked. (*E* ) avGFP dataset, which measures the fluorescence of all possible combinations of pairs of amino acids at 13 sites. (*Left* ) Main effects and pairwise interactions, which account for 57 and 38% of phenotypic variance, respectively. Values are shown only for one of the two of amino acids in each site. The ten pairwise interactions possible among sites 3, 5, 6, 9, and 10 are outlined. (*Right* ) Crystal structure of avGFP (PDB ID: 3e5w). Spheres, the 13 mutated residues; red, the chromophore and the five residues with the strongest phenotypic contribution.

A benefit of combining the sigmoid link function with RFA is that specific genetic effects can then be expressed in simple terms that are comparable across datasets (Fig. 6A). The sigmoid model describes the observed phenotype of a protein variant as an equilibrium between “functional” and “nonfunctional” states that depends on *s*, the variant’s genetic score; the upper and lower bounds represent ensembles in which the fraction of proteins occupying each state approaches the measurable limits. The relative occupancy of the functional state (the ratio of its occupancy to that of the nonfunctional state) is *e*^*s*^, and its fractional occupancy is (1 + *e*^–*s*^)^–1^. This relationship is analogous to the Boltzmann equation, with *s* taking on the role of –∆*G*, the Gibbs free energy difference between the states, expressed in units of *kT*. When *s* equals 0, the functional and nonfunctional states are equally populated, and the phenotype is midway between the upper and lower bounds. An amino acid that increases the score by 2.3 always causes a ten-fold increase in the relative occupancy of the measurable functional state, corresponding to an apparent ∆∆*G* of –1.4 kcal/mol at 37°C. This relationship holds across proteins, functions, and assay systems, which all display the same scaling relationship between a variant’s genetic score and its phenotype, mediated via the probability of occupying the functional state.

We used this framework to interpret the genetic architecture of several example proteins. First, the CR9114-H3 dataset (Fig. 6B) consists of affinity measurements for binding of hemagglutinin to each of 2^16^ antibodies (all possible combinations of ancestral and derived amino acids at 16 sites that evolved during affinity maturation). The vast majority of variants in this library are at or near the lower bound of detectable binding; as a result, the average genetic score is –7.8, corresponding to just 0.04% occupancy of the measurable functional state (∆*G* of 4.7 kcal/mol). Even the highest genetic score in the entire library is only 2.6 — 93% occupancy of the functional state. Main effects at three key sites explain the most phenotypic variance: Substituting any of these from the ancestral to derived state increases the genetic score by between 4.2 and 5.2, corresponding to an increase in relative occupancy of the functional state by 70- to 180-fold and a ∆∆*G* of 2 to 3 kcal/mol each. Other sites make modest contributions: The five next-largest effects each change the genetic score by about 1 (0.7 kcal/mol) when mutated back to the ancestral state, shifting the relative occupancy by 36% each, but reducing the absolute occupancy to just 8% when all five change together. There is virtually no specific epistasis in this genetic architecture (Fig. S1). This means that there are many different combinations of the five moderate-effect sites that provide a sufficient genetic score to confer measurable affinity, but only if the derived state at all three large-effect sites are present. The remaining eight sites have negligible effects on binding and are completely degenerate among functional antibodies.

Second, the genetic architecture of specificity in a protein can be understood by analysis of genetic scores with different substrates (Fig. 6C). A deep mutational scan was performed on the ParD3 protein (20 states at 3 sites in the binding interface) for binding to its cognate ligand ParE3 and a noncognate ligand ParE2 (41). In both cases, first-order determinants account for the vast majority of genetic variance, with main effects on genetic score ranging from strongly positive (3.6) to strongly negative (–4.8); this corresponds to changes in ∆*G* on the order of –2 to 3 kcal/mol and changes in relative occupancy ranging from a 36-fold increase to 120-fold decrease. Effects on specificity can be quantified as the difference in a state’s effect on genetic score for the two substrates. Eight different states distributed across the three variable sites change the genetic score in favor of one ligand or the other by more than 1.6, meaning that they change the relative occupancy of the two substrates by at least 5-fold each (Fig. 6D). For example, the states in the wild-type ParD3 (Asp [D], Lys [K], and Glu [E] in the three variable sites) increase specificity for the cognate ligand by scores of 2.8, 2.7, and 2.2, respectively, corresponding to a 10-fold change in relative occupancy by each; two of these states (1D and 2K) achieve this by increasing the affinity for both ligands with a stronger effect on cognate versus noncognate binding, whereas 3E increases cognate binding but reduces noncognate binding.

Finally, RFA can be used to characterize the scale of epistatic effects on function. In the avGFP dataset (13), pairwise interactions account for 38% of phenotypic variance. Out of 13 sites analyzed, however, main and pairwise effects involving just five sites account for the vast majority of the variance explained (Fig. 6E). These sites, which tightly surround the chromophore in the avGFP crystal structure, engage in a dense network of epistatic interactions in which nine of the ten possible pairwise interactions are non-zero. Although only three of these effects are strong (changing the genetic score by > 1), the total impact is substantial: A total change in genetic score of 2.8 caused by main effects and 7.5 by pairwise interactions, corresponding to 16- and 1,700-fold increases in the relative occupancy of functional state (1.7 and –4.5 kcal/mol), respectively.

## Discussion

Our finding that main and pairwise interactions account for virtually all genetic variation within proteins contrasts with many earlier reports (1–7). This difference is likely attributable to overestimation of epistasis in prior studies, the vast majority of which used reference-based analysis and/or have not fully decoupled specific epistasis from global nonlinearity in the genotype-phenotype relationship. It is possible that higher-order epistasis is more important in some other proteins not examined here, but this seems unlikely, given the consistency of the pattern we observed across 20 different deep mutational scans in a wide variety of proteins with different architectures and functions. Most of the studies we examined focused on a small or moderate number of sites selected a priori because they vary between two functional proteins of interest or they are in important structural positions (e.g., at binding interfaces or active sites). In some cases the sites are clustered, and in others they are dispersed across the protein structure. We therefore have no reason to expect that the sites examined in the studies we analyzed are depleted for higher-order epistasis.

Our analyses assessed the genetic architecture of a single function per protein, rather than the determinants of functional specificity when multiple functions are measured. It is possible that higher-order interactions could be more important in determining functional specificity. Reference-free analysis could easily be expanded to identify the genetic architecture of specificity using DMS studies of multiple functions; a recent study used a similar approach and found that higher-order interactions within a transcription factor are unimportant for determining its specific preferences among DNA binding sites (42). Higher-order epistasis might be more important among loci than it is within proteins, but this is an open question. It is not obvious, for example, that contacts across interfaces between molecules should produce more higher-order genetic interactions than the physically similar contacts that occur within proteins, or that dependencies among molecules in signal transduction or metabolic pathways should involve more higher-order interactions than within the complex environment of a single protein, once the global nonlinearities imposed by these pathways are accounted for.

The lack of higher-order epistasis within proteins may seem surprising, given the complexity of proteins’ three-dimensional structure, in which clusters of three or more residues often contact each other directly. Our findings suggest that the effects of most such clusters can be largely explained by the sum of the pairwise interactions they comprise. But these couplings themselves depend on conformation, which itself is determined by the state at other sites; if a mutation alters the conformation, it will change some pairwise couplings and produce higher-order epistasis. In the datasets we examined, this kind of conformational epistasis appears to be relatively unimportant. A possible explanation is that in these experiments the majority of sites–and therefore presumably the protein’s overall fold–were held constant. Ultimately, the folding of a protein into its native conformation and the couplings that result would seem to require higher-order interactions, and these might be revealed if a large scan of a protein that can adopt multiple conformations were possible. The importance of these interactions in the overall sequence-function relationship relative to lower-order effects, however, is an open question.

The effectiveness of the Boltzmann-like sigmoid function to model nonspecific epistasis seems surprising, because nonlinearity in the genotype-phenotype relationship almost certainly arises from complex biological and technical causes that vary among proteins, functions, and measurement techniques. Our analyses indicate that upper and lower bounds on the dynamic range over which a phenotype can be produced and measured are the primary cause of nonspecific epistasis within proteins. Whether or not the sigmoid transformation is “true,” our findings indicate that accounting for this form of nonlinearity–irrespective of the factors that produce it–is sufficient to allow a low-order model of specific epistasis to provide a parsimonious explanation of genetic architecture that captures virtually all phenotypic variation across all the proteins we examined.

Our finding that RFA outperforms RBA in providing a compact and accurate characterization of the global genotype-phenotype map does not mean that RBA is never useful. There are some settings in which the object of interest is not a protein’s genetic architecture but particular interactions among mutations in the sequence neighborhood immediately around a designated wild-type or ancestral protein. In these cases RBA is appropriate, but it should be used with caution because of its propensity to infer spurious interactions as distance from the reference sequence increases.

Epistasis can make evolutionary trajectories contingent on the chance occurrence of permissive and restrictive epistatic modifiers (27, 43, 44). It was recently shown that the effects of most mutations drift gradually as proteins accumulate substitutions over long-term evolutionary time (45). Our results imply that this drift is likely attributable to the cumulative effect of many small pairwise interactions rather than higher-order modulations. The relative unimportance of high-order epistasis implies that the pairwise dependencies that make evolution contingent on prior mutations are likely to remain largely stable over evolutionary time, rather than being idiosyncratically rewired with every substitution that occurs at other sites.

For scientists who would like to understand how proteins work, our findings are reassuring, but they clarify a major challenge ahead. Proteins’ genetic architecture is intelligible; a small fraction of main and pairwise effects provides a compact and efficient explanation of 90 to 95% of functional genetic variation across the vast space of possible sequences. Complete combinatorial experiments are intractable for many states at more than a few sites or even two states at a moderate number of sites, but the unimportance of high-order epistasis means that it is unnecessary to assay the vast array of triplets, quartets, and so on. The challenge is that the small set of key first- and second-order determinants cannot be efficiently identified from a random sample of variants, because sequence space is huge and most random polypeptides are virtually nonfunctional-particularly when the dynamic range of measurement is limited-so they do not provide useful quantitative information about the sequence states and pairs that they contain. Assessing low-order effects in a single sequence neighborhood is not sufficient, because the resulting estimates would be subject to the same kind of errors and idiosyncracies that plague reference-based estimates. An effective strategy may therefore be to perform comprehensive single- and double-mutant scans using a diverse set of functional proteins as starting points, and then analyze the results using RFA. A critical issue is to determine just how diverse the proteins used as starting points must be, while continuing to improve the efficiency and dynamic range of experimental methods. The potential power of a relatively practical strategy like this has been overlooked to date, presumably because protein architecture is not nearly as complex as it was previously thought to be.

## Methods

### Reference-free analysis (RFA).

Here we define RFA and state its key properties. A detailed exposition with proofs is provided in SI Appendix, and scripts and tutorials for performing RFA are available on GitHub (github.com/whatdoidohaha/RFA).

We consider a simple genotype space defined by *q* states at each of *n* sites, but RFA can also be applied when the number of states varies among sites. Let ***g*** denote a genotype, *y*(***g***) its phenotype, and *G* the set of all genotypes. The global average phenotype is denoted

$$e_{0}=\langle y|G\rangle ,$$

where the brackets indicate averaging of *y* across *G*. RFA decomposes the phenotype into the contribution of individual states and their interactions. The first-order effect of state *s* at site *i* is the difference between the average phenotype of the subset of genotypes sharing that state (denoted $G_{i}^{s}$) and the global average:

$$e_{i}\left(s\right)=\langle y|G_{i}^{s}\rangle -e_{0}.$$

The pairwise interaction between states $s_{1}$ and $s_{2}$ at sites $i_{1}$ and $i_{2}$ is the difference between the average phenotype of the subset of genotypes sharing that state-pair ($G_{i_{1},i_{2}}^{s_{1},s_{2}}$) and the global average after accounting for the main effects:

$$e_{i_{1},i_{2}}\left(s_{1},s_{2}\right)=\langle y|G_{i_{1},i_{2}}^{s_{1},s_{2}}\rangle -\left[e_{0}+e_{i_{1}}\left(s_{1}\right)+e_{i_{2}}\left(s_{2}\right)\right].$$

Similarly, higher-order effects are the difference between the average phenotype of the subset of genotypes sharing a particular set of states and the global average after accounting for the lower-order effects.

RFA predicts the phenotype of a genotype of interest by summing the effects of the states present in that genotype. For a genotype with state *g*_*i*_ in each site *i*, the predicted phenotype under RFA of order *k* is

$$\begin{array}{l} y_{k}\left(\text{g}\right)=e_{0}+\sum_{i}e_{i}\left(g_{i}\right)+\sum_{i_{1}<i_{2}}e_{i_{1}<i_{2}}\left(s_{1},s_{2}\right)+\ldots +\\ \;\sum_{i_{1}<\ldots <i_{k}}e_{i_{1},\ldots ,i_{k}}\left(g_{i_{1}},\ldots ,g_{i_{k}}\right). \end{array}$$

The overall accuracy of this prediction can be quantified by the sum of squared errors:

$$\epsilon_{G}={\sum_{\text{g}\in G}\left[y\left(\text{g}\right)-y_{k}\left(\text{g}\right)\right]}^{2}.$$

Among all possible ways of predicting the phenotype using effects of order up to *k*—including reference-based analysis under any choice of reference genotype and background-averaged analysis under any choice of reference states—RFA minimizes *ϵ*_*G*_ for any *k* for any genetic architecture. For example, when *k* equals zero—that is, when all phenotypes are predicted by a single number—*ϵ*_*G*_ is minimized by the global average phenotype, which is the RFA zero-order term. By explaining as much phenotypic variance as possible at any order of approximation, RFA provides the simplest description of genetic architecture.

A key task in the analysis of genetic architecture is to quantify the contribution of individual states and interactions to the phenotype. RFA facilitates this task by decomposing the total phenotypic variance into the contribution of each factor:

$$Var\left(y|G\right)\left(=\frac{1}{q^{n}}\sum_{\text{g}\in G}{\left[y\left(\text{g}\right)-\langle y|G\rangle \right]}^{2}\right)=\sum_{e\neq e_{9}}\frac{e^{2}}{q^{O\left(e\right)}},$$

where *e* denotes an effect, *O*(*e*) its order, and the summation involves all nonzero-order effects. An effect of order *k* affects the phenotype of one in every *q*^*k*^ genotypes. The expression above therefore states that the amount of phenotypic variance attributable to an effect is the square of its magnitude, normalized by the fraction of genotypes it affects.

A corollary of the definition of reference free effects is that the first-order effects of all states at a site sum to zero:

$$\sum_{1\leq s\leq q}e_{i}\left(s\right)=0.$$

We call this the zero-mean property. The second-order effects of all state-pairs in one site-pair also sum to zero, as do all higher-order effects at a combination of sites.

### Inferring reference-free effects from noisy and incomplete data.

When individual phenotypes are subject to measurement error of variance *ω*, reference-free effects of order *k* computed from these measurements have an error of variance

$$\frac{{\left(q-1\right)}^{k}}{q^{n}}\omega .$$

By definition k≤n, so the variance of computed effects is always less than *ω* and is miniscule when *k* is small relative to *n*. Therefore, reference-free effects can be robustly determined from noisy phenotypic measurements, thanks to the averaging of effects over large numbers of genotypes. By contrast, the error associated with reference-based effects of order *k* is 2^*k*^*ω*, which is always greater than *ω* and typically too large to distinguish effects from errors when *k* > 2. The error associated with background-averaged effects of order *k* is (2*q*)^*k*^*/q*^*n*^ *× ω*, which is greater than the error for reference-free effects of the same order and exceeds *ω* as *k* increases.

When measurement is incomplete, reference-free effects can be inferred by regression. To infer the effects in a truncated model that contains terms of order up to *k*, we model

$$y\left(\text{g}\right)=y_{k}\left(\text{g}\right)+\epsilon \left(\text{g}\right),$$

where the error *ϵ*(***g***) is the sum of all higher-order effects and measurement noise. Regression estimates are obtained by minimizing the sum of squared errors across the set of sampled genotypes (*G*^∗^):

$$\epsilon_{G*}=\sum_{g\in G*}{\left[y\left(\text{g}\right)-y_{k}\left(\text{g}\right)\right]}^{2}.$$

Because RFA minimizes the sum of squared errors across all genotypes, the regression estimates converge to the true values as more genotypes are sampled. Furthermore, the regression estimates are unbiased provided that genotypes are randomly missing. This is because *ϵ*(***g***) is unbiased—equals zero when averaged across all genotypes. This in turn derives from the zero-mean property, which implies that the net phenotypic contribution of any order of effects is zero when averaged across all genotypes; unmodeled higher-order interactions do not bias the regression because they appear as noise to lower-order models.

### Nonspecific epistasis.

We account for nonspecific epistasis by assuming that the effects of sequence states are transformed by a global link function into the observed phenotype (25). The net effect of the sequence states in a genotype is referred to as its genetic score (*s*). We model the link function by a sigmoid that is defined by two parameters, *L* and *U*, which represent the lower and upper bound of phenotype:

$$y\left(\text{g}\right)=L+\frac{U-L}{1+e^{-s\left(\text{g}\right)}}.$$

### Implementation.

Reference-free effects and nonspecific epistasis were jointly inferred by L1-regularized regression. The optimal L1 penalty was determined by maximizing the out-of-sample *R*^2^ in cross-validation. Except for four datasets, cross-validation was performed by randomly partitioning genotypes into training and test sets. For the three datasets with 48 or fewer genotypes and the CR9114-B dataset where only 81 genotypes are above the lower phenotype bound, cross-validation was performed by leaving out each measurement replicate in turn. The R package *lbfgs* was used for numerical optimization. All scripts for inference and analysis are available on GitHub (github.com/whatdoidohaha/RFA).

To jointly infer reference-based effects and nonspecific epistasis, we devised a two-step approach. This was necessary because reference-based analysis is incompatible with regression. For example, regression infers a first-order model by assigning values to the effects of point mutations that best predict the phenotype for both point and higher-order mutants. However, the effect of a point mutation is defined solely by the phenotype of the one variant that contains only that mutation; the regression estimate can be far from true depending on the exact phenotypes of higher-order mutants. For each candidate set of nonspecific epistasis parameters, we computed the reference-based effects on genetic score that exactly recapitulate the phenotypes of mutants up to model order. The effects were then used to predict the phenotype for higher-order mutants. We only predicted higher-order mutants for which all relevant lower-order effects are measured; for example, when a point mutant is missing, any double or higher-order mutant involving that mutation was excluded from prediction. This procedure was repeated for different values of nonspecific epistasis parameters, resulting in values that maximize the *R*^2^.

Background-averaged analysis was originally developed only for binary state spaces. To implement it for spaces with more than two states per site, we extended the recursive matrix formalism of ref. (23) and implemented it in a custom R script. The same multi-state formalism has been independently derived and published recently (34).

### Combinatorial mutagenesis datasets.

We systematically mined the literature for mutagenesis experiments with a combinatorially complete design. Among the many datasets comprising fewer than 100 genotypes, we chose three datasets where high-order epistasis has been reported. Any larger dataset in which precise measurement (*r*^2^ > 0.9 between replicates) is available for at least 40% of possible genotypes was included for analysis. Several datasets were edited as follows.

The methyl-parathion hydrolase activity (46) was measured in the presence of seven different metal cofactors. In every case, second-order reference-free analysis coupled with the sigmoid model of nonspecific epistasis explained more than 90% of phenotypic variance. Only the Ni^2+^ dataset, in which epistasis accounts for the greatest fraction of phenotypic variance, is presented here.

The original dihydrofolate reductase dataset (3) includes a noncoding mutation for a total of 96 variants. We only analyzed the 48 coding site variants fixed for the mutant state in the noncoding site. IC_75_—the antibiotics concentration that reduces the growth rate by 75%—was reported in logarithmic scale, set arbitrarily as –2 when the variant is unviable at any concentration. We reverted the logarithm, making IC_75_ equal to 0 when the variant is unviable.

The hemagglutinin study (39) characterized an identical set of genetic variants in six different genetic backgrounds. We only analyzed the genetic background for which the measurement is most precise (Bei89).

In the avGFP dataset (13), fluorescence is systematically higher in the second measurement replicate by a factor of 1.31. This difference was normalized when combining the two replicates.

The ParB study (47) measures how the transcription factor ParB binds to two DNA motifs, parS and NBS. Because measurement *r*^2^ is less than 0.9 for the NBS dataset, only the parS dataset was analyzed. The absolute fitness of each variant was inferred by comparing the read count before and after the bulk competition assay. Variants with the pre-competition read count fewer than 15 were excluded, resulting in 42.2% coverage of the 160,000 possible genotypes—down from 97.0% in the original study.

The extent of measurement noise in the GB1 dataset (10) could not be directly determined because measurement was not replicated, but comparison to an independent dataset for a subset of variants showed that measurement *r*^2^ is greater than 0.9. Variants with a pre-competition read count fewer than 100 were excluded, resulting in 68.6% coverage of the 160,000 possible genotypes—down from 93.4% in the original study.

## Supplementary Material

Supplement 1

## Figures and Tables

**Table 1.** Combinatorial mutagenesis datasets analyzed in this study.

Protein	Genotype space	Phenotype	Ref.
Methyl-parathion hydrolase	2^5^ (32)	Catalytic activity	(46)
*β*-lactamase	2^5^ (32)	Antibiotics resistance (MIC)	(48)
Dihydrofolate reductase	3 *×* 2^4^ (48)	Antibiotics resistance (IC_75_)	(3)
Influenza A H3N2 hemagglutinin	2^2^ *×* 3^2^ *×* 4^2^ (576)	Viral replication fitness	(39)
Antibody CR6261	2^11^ (2,048)	Affinity for influenza hemagglutinin strain H1	(40)
Antibody CR6261	2^11^ (2,048)	Affinity for influenza hemagglutinin strain H9	(40)
Bacterial antitoxin ParD3	20^3^ (8,000)	Fitness conferred by binding to toxin ParE3	(41)
Bacterial antitoxin ParD3	20^3^ (8,000)	Fitness conferred by binding to toxin ParE2	(41)
Aequorea victoria GFP (avGFP)	2^1^3 (8,192)	Fluorescence	(13)
Bacterial antitoxin ParD3	13 *×* 12 *×* 10 *×* 6 (9,360)	Fitness conferred by binding to toxin ParE3	(49)
Bacterial antitoxin ParD3	13 *×* 12 *×* 10 *×* 6 (9,360)	Fitness conferred by binding to toxin ParE2	(49)
SARS-CoV-2 spike protein	2^15^ (32,768)	Affinity for human ACE2	(7)
Antibody CH65	2^16^ (65,536)	Affinity for influenza hemagglutinin strain MA90	(21)
Antibody CH65	2^16^ (65,536)	Affinity for influenza hemagglutinin strain MA90-G189E	(21)
Antibody CH65	2^16^ (65,536)	Affinity for influenza hemagglutinin strain SI06	(21)
Antibody CR9114	2^16^ (65,536)	Affinity for influenza hemagglutinin strain B	(40)
Antibody CR9114	2^16^ (65,536)	Affinity for influenza hemagglutinin strain H1	(40)
Antibody CR9114	2^16^ (65,536)	Affinity for influenza hemagglutinin strain H3	(40)
Transcription factor ParB	20^4^ (160,000)	Fitness conferred by transcription	(47)
Protein G B1 domain (GB1)	20^4^ (160,000)	Binding enrichment for IgG-Fc	(10)

## Significance Statement

It is widely thought that a protein’s function depends on complex interactions among amino acids. If so, it would be virtually impossible to predict the function of new variants, and understanding how proteins work genetically and biochemically would require huge combinatorial experiments. We show that prior studies overestimated complexity because they analyzed sequence-function relationships from the perspective of a single reference genotype and/or misinterpreted global phenotypic nonlinearities as complex amino acid interactions. By developing a new reference-free approach and using it to reanalyze 20 experimental datasets, we show that additive effects and pairwise interactions alone, along with a simple global nonlinearity, explain the vast majority of functional variation. Higher-order interactions are weak or rare, and a minuscule fraction of possible interactions shape each protein’s function. Our work reveals that protein sequence-function relationships are surprisingly simple and suggests new strategies that are far more tractable than the massive experiments currently used.
