# Supplementary material for: The simplicity of protein sequence-function relationships
Source: bioRxiv. 2023 Sep 5:2023.09.02.556057. Preprint. [Version 1] doi: 10.1101/2023.09.02.556057 (PMC10508729; doi:10.1101/2023.09.02.556057)
Supplement: Supplementary file 1 [file media-1.pdf]

Supporting Information for

The simplicity of protein sequence-function relationships

Yeonwoo Park, Brian P.H. Metzger, and Joseph W. Thornton

Joseph W. Thornton
Email: joet1@uchicago.edu

This PDF file includes:

Figure S1 and S2
Supporting text

Table of Contents

Supplementary figures *1*
Exposition of reference-free analysis *3*
1. Introduction 3
2. Notations 3
3. Definitions and interpretations 3
4. Zero-mean property 5
5. Generalized linear decomposition 6
6. Optimal linear decomposition 7
7. Variance partition 7
8. Robustness to measurement noise 8
9. Orthogonality of orders 8
10. Unbiased estimation by regression 9
11. Relations to other formalisms 10
References 13
Appendix 1. Zero-mean property 15
Appendix 2. Generalized linear decomposition 16
Appendix 3. Optimal linear decomposition 19
Appendix 4. Variance partition 22
Appendix 5. Robustness to measurement noise 25
Appendix 6. Unbiased estimation by regression 28

Supplementary figures

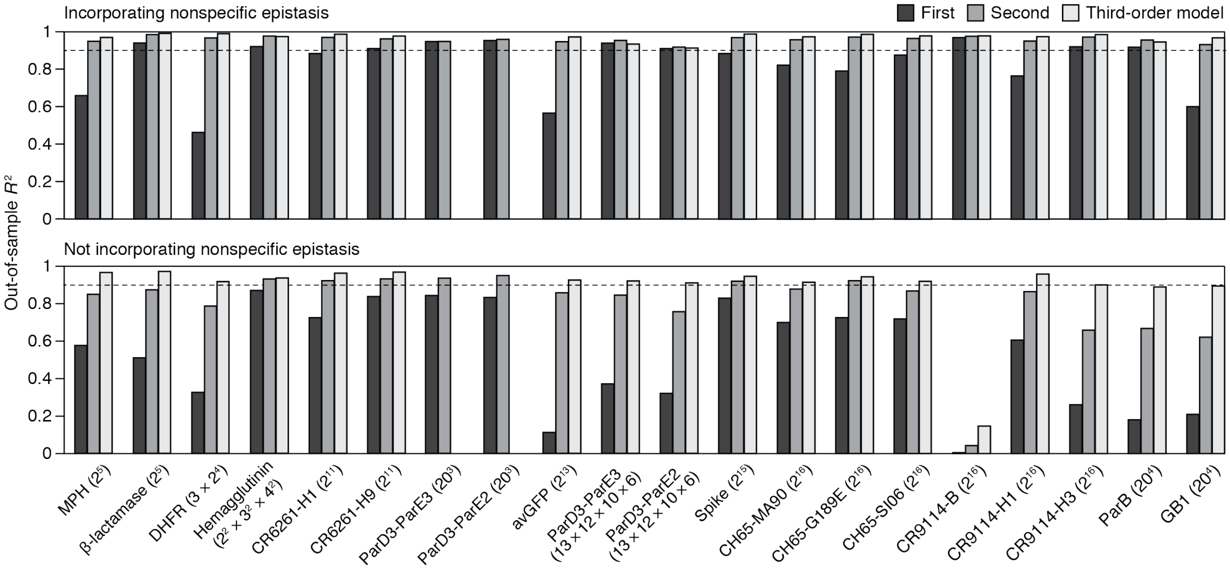

Fig. S1. Reference-free analysis of 20 combinatorial mutagenesis datasets (out-of-sample R^2 in Figs. 2*A* and 3*A*). Datasets are ordered by the total number of genotypes shown in parentheses.

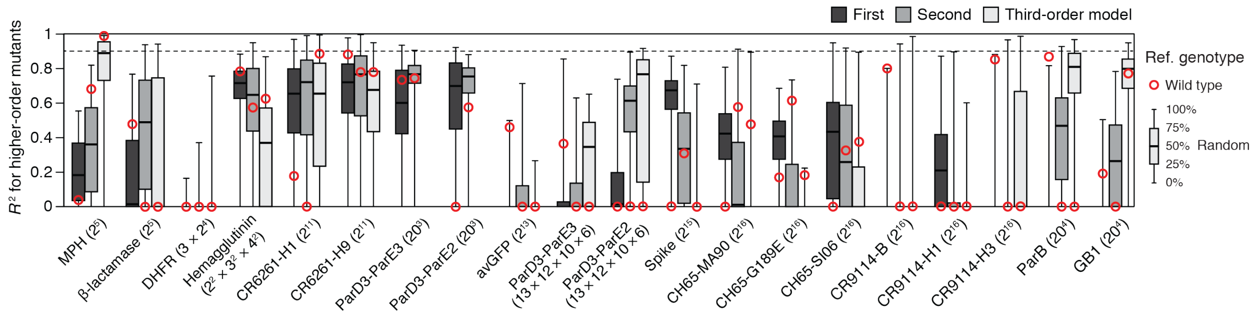

Fig. S2. Reference-based analysis of the 20 combinatorial mutagenesis datasets. Either the published "wild-type" genotype (red circle) or 500 randomly sampled genotypes (boxplot) were used as reference. Each order of model was evaluated by predicting the phenotype of higher-order mutants, accounting for nonspecific epistasis using the sigmoid link function. Negative R^2 values are plotted as zero. Datasets are ordered by the total number of genotypes shown in parentheses.

Exposition of reference-free analysis

1. Introduction

Here we provide a formal exposition of reference-free analysis, including proofs for its key properties. We begin by defining and interpreting reference-free effects. We develop the notion of generalized linear decomposition, a unified formalism for representing any linear decomposition of genetic architecture, which we use to show that reference-free analysis offers the simplest description of genetic architecture—explaining the maximum amount of phenotypic variance that can be explained by any linear decomposition of the same order. Next, we show that the total phenotypic variance can be decomposed into the contribution of each reference-free effect, which enables the variance partition framework for quantifying and comparing the phenotypic contribution of any effect or set of effects. We then show that reference-free effects can be robustly computed from noisy phenotypic measurements and that they can be estimated from an incomplete sample of genotype space by regression. We end by comparing reference-free analysis with other formalisms for decomposing the genetic architecture.

2. Notations

Reference-free analysis can be applied to any discrete-state genetic architecture, but for simplicity we consider a genotype space with the same number of states (q) across sites. The definitions and proofs provided here can be easily extended to genotype spaces with different numbers of states across sites. The n-tuple $\boldsymbol{g} = (g_1, \ldots, g_n)$ denotes a genotype with state g_i in site $i = 1, \ldots, n$. The phenotype of $\boldsymbol{g}$ is written as $y(\boldsymbol{g})$. The set of all genotypes is denoted by G, and the set of all genotypes sharing states $s_1, \ldots, s_k$ in sites $i_1, \ldots, i_k$ is denoted by $G_{i_1,\ldots,i_k}^{s_1,\ldots,s_k}$. Angled brackets denote averaging over a set; for example, $\langle y|G\rangle$ is the average phenotype of all genotypes. The set $N = \{1, \ldots, n\}$ is used to denote iterations over sites and site-combinations; $\Sigma_{i\in N}$ indicates summation over all sites and $\Sigma_{i_1<i_2\in N}$ over all site-pairs. Likewise, $Q = \{1, \ldots, q\}$ is used to denote iterations over states and state-combinations.

We do not consider nonspecific epistasis here. Nonspecific epistasis can be incorporated by modeling $y(\boldsymbol{g})$ as a nonlinear transformation of genetic score $s(\boldsymbol{g})$, which would be defined according to the formalism below.

3. Definitions and interpretations

We first present reference-free analysis as a stepwise approximation of genetic architecture. Two alternative interpretations are then presented.

The intercept or zero-order term, e_0, is defined as the average phenotype of all genotypes:

$$e_0 = \langle y|G\rangle.$$

This is the best single-parameter approximation of genetic architecture in the sense that it minimizes the mean squared error of phenotypic prediction across sequence space.

The first-order term representing state s in site i, denoted by $e_i(s)$, is defined as

$$e_i(s) = \langle y|G_i^s\rangle - e_0.$$

It can be considered the error associated with approximating $\langle y|G_i^s\rangle$—the average phenotype of the subspace comprising all genotypes sharing state s in site i—by the lower-order term e_0 (the average phenotype across the entire space).

The above expression extends naturally to higher-order terms. The second-order term for the state-pair (s_1, s_2) in site-pair (i_1, i_2), denoted by $e_{i_1,i_2}(s_1, s_2)$, is the error associated with approximating $\left\langle y\middle|G_{i_1,i_2}^{s_1,s_2}\right\rangle$ using the lower-order terms:

$$e_{i_1,i_2}(s_1, s_2) = \left\langle y\middle|G_{i_1,i_2}^{s_1,s_2}\right\rangle - \left[e_0 + e_{i_1}(s_1) + e_{i_2}(s_2)\right].$$

In general, the k-th-order term for the state-combination (s_1, …, s_k) in site-combination (i_1, …, i_k), denoted by $e_{i_1,\ldots,i_k}(s_1, \ldots, s_k)$, is defined as

$$e_{i_1,\ldots,i_k}(s_1, \ldots, s_k) = \left\langle y\middle|G_{i_1,\ldots,i_k}^{s_1,\ldots,s_k}\right\rangle - \left\langle y\middle|G_{i_1,\ldots,i_k}^{s_1,\ldots,s_k}\right\rangle_{(k-1)}, \quad (1)$$

where the subscript (k – 1) indicates approximation by terms of order up to (k – 1):

$$\left\langle y\middle|G_{i_1,\ldots,i_k}^{s_1,\ldots,s_k}\right\rangle_{(k-1)} = e_0 + \sum_{\alpha\in K} e_{i_\alpha}(s_\alpha) + \sum_{\alpha_1<\alpha_2\in K} e_{i_{\alpha_1},i_{\alpha_2}}(s_{\alpha_1}, s_{\alpha_2}) + \cdots + \sum_{\alpha_1<\cdots<\alpha_{k-1}\in K} e_{i_{\alpha_1},\ldots,i_{\alpha_{k-1}}}(s_{\alpha_1}, \ldots, s_{\alpha_{k-1}}),$$

where K denotes the set $\{1, \ldots, k\}$.

This stepwise process builds an increasingly refined approximation of genetic architecture. The zero-order term is the crudest approximation, a single average approximating the entire genotype space. The first-order terms describe the average phenotypes of nq subspaces, each consisting of all genotypes that share a particular state in a particular site. The second-order terms describe the average phenotypes of smaller subspaces, each consisting of all genotypes sharing a particular pair of states in a particular pair of sites. Higher-order terms offer finer descriptions, with the n-th-order terms describing the phenotypes of the smallest subspaces–individual genotypes.

We have so far presented reference-free terms as errors associated with lower-order approximations. They can also be interpreted as phenotypic effects. For example, the first-order term $e_i(s)$ quantifies how the average phenotype of all genotypes sharing the state s in site i differs from that of all genotypes; it can thus be interpreted as the average phenotypic effect of state s in site i. Similarly, the second-order term $e_{i_1,i_2}(s_1, s_2)$ quantifies how the average phenotype of all genotypes sharing the state-pair (s_1, s_2) in site-pair (i_1, i_2) differs from that of all genotypes after accounting for the first-order effects; it is therefore the average epistatic effect of the state-pair.

A third perspective on reference-free analysis interprets the terms of order k as measuring the context-dependence of terms of order $k-1$. Let us re-think the definition of the first-order term

$$e_i(s) = \langle y|G_i^s\rangle - e_0.$$

$\langle y|G_i^s\rangle$ can be considered the intercept for a genotype space consisting only of genotypes with state s in site i. We denote this relationship by $\langle y|G_i^s\rangle = e_0|_i^s$. Thus,

$$e_i(s) = e_0|_i^s - e_0.$$

In this expression, $e_i(s)$ quantifies how different the intercept is when calculated for the complete space G versus the subspace G_i^s. A parallel exists for second-order terms:

$$\begin{aligned} e_{i_1,i_2}(s_1,s_2) &= \left\langle y\middle|G_{i_1,i_2}^{s_1,s_2}\right\rangle - \left[e_0 + e_{i_1}(s_1) + e_{i_2}(s_2)\right] \\ &= \left\langle y\middle|G_{i_1,i_2}^{s_1,s_2}\right\rangle - \left\langle y\middle|G_{i_1}^{s_1}\right\rangle - \left\langle y\middle|G_{i_2}^{s_2}\right\rangle + \langle y|G\rangle \\ &= \left[\left\langle y\middle|G_{i_1,i_2}^{s_1,s_2}\right\rangle - \left\langle y\middle|G_{i_1}^{s_1}\right\rangle\right] - \left[\left\langle y\middle|G_{i_2}^{s_2}\right\rangle - \langle y|G\rangle\right] \\ &= e_{i_2}(s_2)|_{i_1}^{s_1} - e_{i_2}(s_2), \end{aligned}$$

where $e_{i_2}(s_2)|_{i_1}^{s_1}$ is the first-order term $e_{i_2}(s_2)$ for the subspace $G_{i_1}^{S_1}$. In general,

$$e_{i_1,\ldots,i_k}(s_1,\ldots,s_k) = e_{i_2,\ldots,i_k}(s_2,\ldots,s_k)|_{i_1}^{s_1} - e_{i_2,\ldots,i_k}(s_2,\ldots,s_k).$$

Here site i_1 is chosen as the context but any other site could be chosen instead.

Finally, the phenotype of any particular genotype is given by the sum of all relevant reference-free terms: Substituting n for k in Eq. (1) and noting that $\left\langle y\middle|G_{i_1,\ldots,i_n}^{g_1,\ldots,g_n}\right\rangle$ is simply the phenotype of $\boldsymbol{g} = (g_1, \ldots, g_n)$, it follows that

$$y(\boldsymbol{g}) = e_0 + \sum_{i\in N} e_i(g_i) + \sum_{i_1<i_2\in N} e_{i_1,i_2}(g_{i_1},g_{i_2}) + \cdots + \sum_{i_1<\cdots<i_k\in N} e_{i_1,\ldots,i_k}(g_{i_1},\ldots,g_{i_k}) + \cdots + e_{i_1,\ldots,i_n}(g_1,\ldots,g_n). \quad (2)$$

4. Zero-mean property

Reference-free terms satisfy an equality that we call the zero-mean property. This property forms the basis of all desirable attributes of reference-free analysis, and can be said to define reference-free analysis in the sense described below. We simply state the property here and prove it in Appendix 1.

For first-order terms, the zero-mean property states that the mean of all q terms at a site is zero:

$$\langle e_i | Q \rangle = \frac{1}{q} \sum_{s \in Q} e_i(s) = 0,$$

where Q denotes the set of all q states. This holds for any genetic architecture by virtue of the definition of reference-free terms. For conciseness, let • denote the averaging of effects across all states. For example, $e_i(\cdot)$ is the average of the q first-order effects for site i, and $e_{i_1,i_2}(\cdot, s_2)$ is the average of the q second-order effects for site-pair (i_1, i_2) that share state s_2 in site i_2. The zero-mean property for first-order effects can be restated as $e_i(\cdot) = 0$. In general, for any site-combination (i_1, …, i_k), the mean of any q effects that vary across a single site is zero:

$$e_{i_1,\dots,i_k}(\cdot, s_2, \dots, s_k) = e_{i_1,\dots,i_k}(s_1, \cdot, s_3, \dots, s_k) \dots = e_{i_1,\dots,i_k}(s_1, \dots, s_{k-1}, \cdot) = 0,$$

where (s_1, …, s_k) can be any state-combination. When the q^2 second-order effects for a site-pair are arranged in a $q \times q$ matrix, the zero-mean property means that every row and column of the matrix sums to zero. Similarly, when the q^3 third-order effects for a site-triplet are arranged in a $q \times q \times q$ array, every one-dimensional section (and thus every two-dimensional section and the entire array) sums to zero.

5. Generalized linear decomposition

Reference-free analysis linearly decomposes the phenotype into the effect of each state and state-combination. There are alternative ways of linearly decomposing the phenotype, including reference-based and background-averaged analyses. We now show that reference-free analysis explains the maximum amount of phenotypic variance that can be explained by any linear decomposition of the same order. Proving this requires a common formalism to express any linear decomposition of a genetic architecture and showing that reference-free analysis is the most powerful. We call this unified formalism the generalized linear decomposition.

Eq. (2), considered without the reference-free definition of the terms, states a general formula for linearly decomposing the phenotype. Let us count the number of terms in this decomposition. There are nq first-order terms, q for each site. There are $\binom{n}{2}q^2$ second-order terms—q^2 pairs of states for every possible pair of sites. With $\binom{n}{k}q^k$ terms of order k, the total number of terms sums to $(q + 1)^n$, which is greater than the total number of genotypes. This means that there are many ways of linearly decomposing the same genetic architecture, distinguished by how exactly the terms in Eq. (2) are defined. Constraining the terms to satisfy the zero-mean property yields the reference-free decomposition: the q first-order terms for a site must sum to zero, reducing their degrees of freedom to $(q - 1)$; every row and column of the $q \times q$ matrix of q^2 second-order terms for a site-pair must sum to zero, reducing their degrees of freedom to $(q - 1)^2$. In general, the zero-mean property reduces the degrees of freedom of k-th-order terms to $\binom{n}{k}(q-1)^k$, which sum across k to q^n. This is the sense in which the zero-mean property defines reference-free decomposition.

Alternative constraints yield alternative decompositions. For example, given a genotype $\boldsymbol{r}$ = (r_1, …, r_n), setting every term involving the state r_i in site i to zero yields the familiar reference-based decomposition with $\boldsymbol{r}$ as the reference genotype.

We call Eq. (2) the generalized linear decomposition of genetic architecture. In Appendix 2, we show that any linear decomposition of genetic architecture is a special case of Eq. (2) obtained by subjecting the terms to a set of constraints that reduces their degrees of freedom to q^n. The notion of generalized linear decomposition allows us to ask the following question.

6. Optimal linear decomposition

Among the infinitely many ways of linearly decomposing a genetic architecture, which is the most optimal? Answering this question requires defining optimality. Given a particular linear decomposition, let $y_k(\boldsymbol{g})$ denote the phenotype of $\boldsymbol{g}$ approximated by the terms of order up to k—the truncation of Eq. (2) removing all higher-order terms. The accuracy of this approximation can be measured by the mean squared error:

$$\epsilon_G[y_k] = \frac{1}{q^n} \sum_{\boldsymbol{g} \in G} [y(\boldsymbol{g}) - y_k(\boldsymbol{g})]^2.$$

We define an optimal linear decomposition of order k as that which minimizes ε_G. Appendix 3 shows that the reference-free decomposition minimizes ε_G for every k for any genetic architecture: it offers the most accurate linear approximation to the genetic architecture at every order of approximation. (When k equals n, every linear decomposition is equally exact.)

7. Variance partition

A key task in understanding the causal architecture of any system is to identify the relative contribution of each causal factor to the variation in dependent variable. Intuitively, causal terms with larger coefficients imply greater importance. However, magnitude cannot be the sole criterion because terms of different orders affect different numbers of genotypes. A reference-free effect of order k contributes to the phenotype of 1 in q^k genotypes; given the same magnitude, a lower-order effect is more consequential than a higher-order effect because it influences more genotypes. We therefore need a way to jointly consider the magnitude and order of terms.

Reference-free analysis enables a variance partition framework. This makes possible such statements as "this term explains 5% of total phenotypic variance" or "the first-order terms together explain 80% of total phenotypic variance." The applicability of variance partition is a unique feature of reference-free analysis enabled by its zero-mean property.

The total phenotypic variance

$$Var(y|G) = \frac{1}{q^n} \sum_{\boldsymbol{g} \in G} [y(\boldsymbol{g}) - \langle y|G \rangle]^2$$

quantifies the total amount of phenotypic variation caused by genetic variation. Appendix 4 shows that it can be decomposed into the contribution of each reference-free effect:

$$Var(y|G) = \sum_{e \neq e_0} \frac{e^2}{q^{O(e)}},$$

where e denotes an effect, $O(e)$ its order, and the summation involves all nonzero-order effects. Note that $1/q^{O(e)}$ is the fraction of genotypes whose phenotype involves the effect e. The variance contribution of each effect is therefore the square of its magnitude normalized by the fraction of genotypes it affects. This confirms our intuition that a lower-order effect makes a greater phenotypic contribution than a higher-order effect of the same magnitude.

8. Robustness to measurement noise

We consider how noise in phenotypic measurement impacts the computation of reference-free effects. An experimentally characterized genetic architecture is a superposition of the true architecture and an unstructured architecture where each phenotype is an independent draw from a noise distribution. The reference-free effects of this unstructured architecture are the measurement errors for the true architecture.

Consider an unstructured architecture generated by sampling each phenotype independently from a noise distribution with variance ω. The zero-order effect is the average of q^n independent draws, so its variance is ω/q^n; this is the error associated with the measurement of the true zero-order effect. Appendix 5 continues this calculation to higher orders, showing that the variance associated with an effect of order k is

$$\frac{(q-1)^k}{q^n}\omega.$$

This is always smaller than ω—the noise involved in individual phenotypic measurements—and negligible when k is small relative to n. The reference-free formalism is therefore highly robust to measurement noise.

This is not the case for alternative formalisms. In reference-based analysis, an effect of order k is a sum or subtraction of 2^k phenotypes and therefore the error associated with it is $2^k\omega$. This is always greater than ω and rises exponentially with order such that measurement is practically impossible for all but the lowest few orders. In background-averaged analysis, an effect of order k is computed by averaging the corresponding reference-based effect across all possible genetic backgrounds—of which there are $q^{(n-k)}$ (1). The error associated with it is therefore $2^k/q^{(n-k)} \times \omega$, which equals $(2q)^k/q^n \times \omega$. This is greater than the error associated with reference-free effects of the same order and exceeds ω as k rises.

9. Orthogonality of orders

We now show that each order of reference-free effects is orthogonal to any other order of effects. That is, altering the genetic architecture by changing one order of effects does not change the value of any other order of effects. Here we demonstrate the orthogonality between the first and

other orders of effects. Let $y_{-1}(\boldsymbol{g})$ denote the phenotypic contribution of second- and higher-order effects on the genotype $\boldsymbol{g} = (g_1, \ldots, g_n)$:

$$y_{-1}(\boldsymbol{g}) = \sum_{i_1<i_2 \in N} e_{i_1,i_2}(g_{i_1}, g_{i_2}) + \sum_{i_1<i_2<i_3 \in N} e_{i_1,i_2,i_3}(g_{i_1}, g_{i_2}, g_{i_3}) + \cdots$$

Consider averaging y_{-1} across the subset of genotypes sharing state s in site j:

$$\begin{aligned}\langle y_{-1} | G_j^s \rangle &= \frac{1}{q^{n-1}} \sum_{\boldsymbol{g} \in G_j^s} y_{-1}(\boldsymbol{g}) \\ &= \frac{1}{q^{n-1}} \sum_{\boldsymbol{g} \in G_j^s} \left[\sum_{i_1<i_2 \in N} e_{i_1,i_2}(g_{i_1}, g_{i_2}) + \sum_{i_1<i_2<i_3 \in N} e_{i_1,i_2,i_3}(g_{i_1}, g_{i_2}, g_{i_3}) + \cdots \right] \\ &= 0,\end{aligned}$$

where the last equality follows from the zero-mean property because each order of effects is summed across all states in one or more sites. It then follows that the values of first-order effects are independent of the values of higher-order effects:

$$\begin{aligned}e_j(s) &= \langle y | G_j^s \rangle - e_0 \\ &= \langle y_1 + y_{-1} | G_j^s \rangle - e_0 \\ &= \langle y_1 | G_j^s \rangle + \langle y_{-1} | G_j^s \rangle - e_0 \\ &= \langle y_1 | G_j^s \rangle - e_0,\end{aligned}$$

where y_1 denotes the phenotypic contribution of zero- and first-order effects. The subtraction by e_0 also makes the first-order effect independent of the zero-order effect. Overall, this derivation shows that the pattern of phenotypic variation produced by other orders effects appears as noise to the first-order model.

10. Unbiased estimation by regression

The exact computation of reference-free effects requires phenotyping every genotype. However, it is possible to accurately estimate them from a randomly sampled subset of genotypes. Recall that among all linear decompositions of the same order, reference-free decomposition minimizes the mean squared error across the genotype space. Regression estimates, which minimize the mean squared error across sampled genotypes, thus converge to truth as sample coverage increases. This is why fitting a truncated low-order model to complete data recovers the true low-order effects without erroneously incorporating higher-order interactions.

To formalize this idea, let y_k denote the generalized linear decomposition truncated after order k:

$$y_k(\boldsymbol{g}) = e_0 + \sum_{i \in N} e_i(g_i) + \sum_{i_1<i_2 \in N} e_{i_1,i_2}(g_{i_1}, g_{i_2}) + \cdots + \sum_{i_1<\cdots<i_k \in N} e_{i_1,\ldots,i_k}(g_{i_1}, \ldots, g_{i_k}).$$

The best-fit model $\hat{y}_k$ is found by minimizing the mean squared error across the set of sampled genotypes (G^*):

$$\hat{y}_k = \text{argmin} \sum_{\boldsymbol{g} \in G^*} [y(\boldsymbol{g}) - y_k(\boldsymbol{g})]^2.$$

One problem with this formulation is that, because of the degeneracy of generalized linear decomposition, there are many possible $\hat{y}_k$ that minimize the error. This can be circumvented by performing a constrained regression, in which the terms are constrained to satisfy the zero-mean property. Alternatively, Appendix 6 shows that a simpler two-step procedure yields the same answer: an unconstrained regression is first performed to find any $\hat{y}_k$ and then the zero-mean property is enforced post hoc on the inferred effects. This two-step procedure was used for the analysis of experimental datasets. $\hat{y}_k$ thus obtained is consistent—converges to truth as sample coverage increases—and Appendix 6 shows that it is also unbiased—the expected value of each effect equals the true effect.

11. Relations to other formalisms

We end by discussing the relationship between reference-free analysis and two other formalisms for capturing the global genetic architecture—Fourier analysis (2, 3) and background-averaged epistasis (1, 4).

Fourier analysis. The two available implementations of Fourier analysis—simplex encoding (5) and graph Fourier transform (6)—are more economical than reference-free analysis because they use the minimum necessary number of terms (q^n) to decompose the genetic architecture as opposed to the excess ($[q + 1]^n$) of reference-free analysis. This economy, however, causes the phenotype of each genotype to depend on every model term—resulting in greater sensitivity to missing genotypes—and renders these implementations difficult to intuit. To illustrate this, we consider the genetic architecture of a single nucleotide. Reference-free analysis of this architecture consists of five terms: the intercept e_0 and the first-order effects e(A), e(C), e(G), and e(T). These effects map to phenotype simply as

$$\begin{aligned} y(A) &= e_0 + e(A), \\ y(C) &= e_0 + e(C), \\ y(G) &= e_0 + e(G), \\ y(T) &= e_0 + e(T). \end{aligned}$$

The simplex encoding of the same architecture consists of four terms: the intercept s_0 and the first-order terms s(W), s(Y), and s(K). These terms map to phenotype as

$$\begin{aligned} y(A) &= s_0 + s(W) - s(Y) - s(K), \\ y(C) &= s_0 - s(W) + s(Y) - s(K), \\ y(G) &= s_0 - s(W) - s(Y) + s(K), \\ y(T) &= s_0 + s(W) + s(Y) + s(K). \end{aligned}$$

The simplex terms are related to the reference-free effects as follows:

$$s_0 = e_0,$$
$$s(W) = \frac{e(A)+e(T)}{2},$$
$$S(Y) = \frac{e(C)+e(T)}{2},$$
$$S(K) = \frac{e(G)+e(T)}{2}.$$

Conversely,

$$e(A) = s(W) - s(Y) - s(K),$$
$$e(C) = -s(W) + s(Y) - s(K),$$
$$e(G) = -s(W) - s(Y) + s(K),$$
$$e(T) = s(W) + s(Y) + s(K).$$

Simplex encoding can be considered a compressed encoding of reference-free analysis: the four linearly dependent reference-free effects are encoded by three linearly independent simplex terms. In general, the q^k reference-free effects for a site-combination of order k are compressed into $(q - 1)^k$ simplex terms. The degree of compression can be substantial for high-order effects: in a genotype space consisting of ten sites and two states, there are $2^{10} = 1{,}024$ tenth-order reference-free effects but only $1^{10} = 1$ tenth-order simplex term. However, for low-order effects the degree of compression is minor, especially when there are many states. For proteins with 20 states, the number of simplex terms is 19 for a site as opposed to 20 in reference-free analysis, and 361 for a site-pair as opposed to 400. Furthermore, the dependence of each phenotype on all model terms means that simplex encoding is more sensitive to missing genotypes. Simplex encoding also requires the number of states to be identical across sites, which is violated in many experimental datasets. Graph Fourier transform overcomes this limit using a sophisticated mathematical machinery, but it is expected to share the same sensitivity to missing genotypes.

Background-averaging. Background-averaged analysis is a modification of reference-based analysis, in which mutational effects are measured from a designated reference state to other states but are averaged across all genetic backgrounds. We illustrate it on a genotype space consisting of three states, denoted by 0, 1, and 2, in two sites. We denote the phenotype of a genotype with state a in site 1 and b in site 2 simply as y_{ab}, and choose state 0 as the reference state to calculate the effects of mutations to states 1 and 2.

The intercept is defined as the average phenotype of all genotypes:

$$b_0 = \bar{y}.$$

The first-order effect of state 1 in site 1, denoted by $b_{1\cdot}$, is the effect of mutating state 0 to 1 at site 1 measured on the background of each possible state in site 2:

$$b_{1\cdot} = \frac{(y_{10}-y_{00})+(y_{11}-y_{01})+(y_{12}-y_{02})}{3}.$$

Likewise, the first-order effect of state 1 in site 2, denoted by $b_{\cdot 1}$, is the effect of mutating state 0 to 1 at site 2 measured on the background of each possible state in site 1:

$$b_{\cdot 1} = \frac{(y_{01}-y_{00})+(y_{11}-y_{10})+(y_{21}-y_{20})}{3}.$$

Because there are only two sites in this example, there is no genetic background over which to average pairwise interactions. Therefore, background-averaged interactions are identical to the corresponding reference-based interactions. For example,

$$b_{11} = y_{11} - y_{10} - y_{01} + y_{00}.$$

These definitions can be stated compactly as matrix multiplication:

$$\begin{pmatrix} b_0 \\ b_{\cdot 1} \\ b_{\cdot 2} \\ b_{1\cdot} \\ b_{11} \\ b_{12} \\ b_{2\cdot} \\ b_{21} \\ b_{22} \end{pmatrix} = \begin{pmatrix} \frac{1}{9} & \frac{1}{9} & \frac{1}{9} & \frac{1}{9} & \frac{1}{9} & \frac{1}{9} & \frac{1}{9} & \frac{1}{9} & \frac{1}{9} \\ -\frac{1}{3} & \frac{1}{3} & 0 & -\frac{1}{3} & \frac{1}{3} & 0 & -\frac{1}{3} & \frac{1}{3} & 0 \\ -\frac{1}{3} & 0 & \frac{1}{3} & -\frac{1}{3} & 0 & \frac{1}{3} & -\frac{1}{3} & 0 & \frac{1}{3} \\ -\frac{1}{3} & -\frac{1}{3} & -\frac{1}{3} & \frac{1}{3} & \frac{1}{3} & \frac{1}{3} & 0 & 0 & 0 \\ 1 & -1 & 0 & -1 & 1 & 0 & 0 & 0 & 0 \\ 1 & 0 & -1 & -1 & 0 & 1 & 0 & 0 & 0 \\ -\frac{1}{3} & -\frac{1}{3} & -\frac{1}{3} & 0 & 0 & 0 & \frac{1}{3} & \frac{1}{3} & \frac{1}{3} \\ 1 & -1 & 0 & 0 & 0 & 0 & -1 & 1 & 0 \\ 1 & 0 & -1 & 0 & 0 & 0 & -1 & 0 & 1 \end{pmatrix} \begin{pmatrix} y_{00} \\ y_{01} \\ y_{02} \\ y_{10} \\ y_{11} \\ y_{12} \\ y_{20} \\ y_{21} \\ y_{22} \end{pmatrix}.$$

The structure of this matrix explains why background-averaged analysis is more sensitive to measurement noise and missing genotypes than is reference-free analysis. To see this, we also represent reference-free analysis as matrix multiplication (for simplicity, effects for state 2 are omitted):

$$\begin{pmatrix} e_0 \\ e_1(0) \\ e_1(1) \\ e_2(0) \\ e_2(1) \\ e_{1,2}(0,0) \\ e_{1,2}(0,1) \\ e_{1,2}(1,0) \\ e_{1,2}(1,1) \end{pmatrix} = \begin{pmatrix} \frac{1}{9} & \frac{1}{9} & \frac{1}{9} & \frac{1}{9} & \frac{1}{9} & \frac{1}{9} & \frac{1}{9} & \frac{1}{9} & \frac{1}{9} \\ \frac{2}{9} & \frac{2}{9} & \frac{2}{9} & -\frac{1}{9} & -\frac{1}{9} & -\frac{1}{9} & -\frac{1}{9} & -\frac{1}{9} & -\frac{1}{9} \\ -\frac{1}{9} & -\frac{1}{9} & -\frac{1}{9} & \frac{2}{9} & \frac{2}{9} & \frac{2}{9} & -\frac{1}{9} & -\frac{1}{9} & -\frac{1}{9} \\ \frac{2}{9} & -\frac{1}{9} & -\frac{1}{9} & \frac{2}{9} & -\frac{1}{9} & -\frac{1}{9} & \frac{2}{9} & -\frac{1}{9} & -\frac{1}{9} \\ -\frac{1}{9} & \frac{2}{9} & -\frac{1}{9} & -\frac{1}{9} & \frac{2}{9} & -\frac{1}{9} & -\frac{1}{9} & \frac{2}{9} & -\frac{1}{9} \\ \frac{4}{9} & -\frac{2}{9} & -\frac{2}{9} & -\frac{2}{9} & \frac{1}{9} & \frac{1}{9} & -\frac{2}{9} & \frac{1}{9} & \frac{1}{9} \\ -\frac{2}{9} & \frac{4}{9} & -\frac{2}{9} & \frac{1}{9} & -\frac{2}{9} & \frac{1}{9} & \frac{1}{9} & -\frac{2}{9} & \frac{1}{9} \\ -\frac{2}{9} & \frac{1}{9} & \frac{1}{9} & \frac{4}{9} & -\frac{2}{9} & -\frac{2}{9} & -\frac{2}{9} & \frac{1}{9} & \frac{1}{9} \\ \frac{1}{9} & -\frac{2}{9} & \frac{1}{9} & -\frac{2}{9} & \frac{4}{9} & -\frac{2}{9} & \frac{1}{9} & -\frac{2}{9} & \frac{1}{9} \end{pmatrix} \begin{pmatrix} y_{00} \\ y_{01} \\ y_{02} \\ y_{10} \\ y_{11} \\ y_{12} \\ y_{20} \\ y_{21} \\ y_{22} \end{pmatrix}.$$

Many of the matrix elements for background-averaged analysis are 1 or –1, indicating that the noise in phenotypic measurement is directly propagated to the effects; the many zeros also mean that the effects are calculated by averaging only over subsets of genotypes. By contrast, all the matrix elements for reference-free analysis are nonzero and smaller than 4/9 in magnitude.

In background-averaged analysis, y_{00} contributes to all nine effects whereas y_{22} contributes to only four. Genotypes containing the reference states contribute disproportionately to the effects, causing background-averaged analysis to be particularly sensitive to noise and missing measurement for these genotypes. By contrast, all genotypes contribute with an equal weight to reference-free effects.

Although background-averaged effects are defined intuitively, their mapping back to phenotype is not. This can be seen by inverting the above matrix multiplication:

$$\begin{pmatrix} y_{00} \\ y_{01} \\ y_{02} \\ y_{10} \\ y_{11} \\ y_{12} \\ y_{20} \\ y_{21} \\ y_{22} \end{pmatrix} = \begin{pmatrix} 1 & -\frac{1}{3} & -\frac{1}{3} & -\frac{1}{3} & \frac{1}{9} & \frac{1}{9} & -\frac{1}{3} & \frac{1}{9} & \frac{1}{9} \\ 1 & \frac{2}{3} & -\frac{1}{3} & -\frac{1}{3} & -\frac{2}{9} & \frac{1}{9} & -\frac{1}{3} & -\frac{2}{9} & \frac{1}{9} \\ 1 & -\frac{1}{3} & \frac{2}{3} & -\frac{1}{3} & \frac{1}{9} & -\frac{2}{9} & -\frac{1}{3} & \frac{1}{9} & -\frac{2}{9} \\ 1 & -\frac{1}{3} & -\frac{1}{3} & \frac{2}{3} & -\frac{2}{9} & -\frac{2}{9} & -\frac{1}{3} & \frac{1}{9} & \frac{1}{9} \\ 1 & \frac{2}{3} & -\frac{1}{3} & \frac{2}{3} & \frac{4}{9} & -\frac{2}{9} & -\frac{1}{3} & -\frac{2}{9} & \frac{1}{9} \\ 1 & -\frac{1}{3} & \frac{2}{3} & \frac{2}{3} & -\frac{2}{9} & \frac{4}{9} & -\frac{1}{3} & \frac{1}{9} & -\frac{2}{9} \\ 1 & -\frac{1}{3} & -\frac{1}{3} & -\frac{1}{3} & \frac{1}{9} & \frac{1}{9} & \frac{2}{3} & -\frac{2}{9} & -\frac{2}{9} \\ 1 & \frac{2}{3} & -\frac{1}{3} & -\frac{1}{3} & -\frac{2}{9} & \frac{1}{9} & \frac{2}{3} & \frac{4}{9} & -\frac{2}{9} \\ 1 & -\frac{1}{3} & \frac{2}{3} & -\frac{1}{3} & \frac{1}{9} & -\frac{2}{9} & \frac{2}{3} & -\frac{2}{9} & \frac{4}{9} \end{pmatrix} \begin{pmatrix} b_0 \\ b_{\cdot 1} \\ b_{\cdot 2} \\ b_{1\cdot} \\ b_{11} \\ b_{12} \\ b_{2\cdot} \\ b_{21} \\ b_{22} \end{pmatrix}.$$

Each phenotype is a weighted sum of all background-averaged effects, including those that represent states and state-combinations not in the genotype of interest. The error in the estimate of an effect is therefore propagated to every genotype. By contrast, the mapping from reference-free effects to phenotype is simple and sparse:

$$\begin{pmatrix} y_{00} \\ y_{01} \\ y_{10} \\ y_{11} \end{pmatrix} = \begin{pmatrix} 1 & 1 & 0 & 1 & 0 & 1 & 0 & 0 & 0 \\ 1 & 1 & 0 & 0 & 1 & 0 & 1 & 0 & 0 \\ 1 & 0 & 1 & 1 & 0 & 0 & 0 & 1 & 0 \\ 1 & 0 & 1 & 0 & 1 & 0 & 0 & 0 & 1 \end{pmatrix} \begin{pmatrix} e_0 \\ e_1(0) \\ e_1(1) \\ e_2(0) \\ e_2(1) \\ e_{1,2}(0,0) \\ e_{1,2}(0,1) \\ e_{1,2}(1,0) \\ e_{1,2}(1,1) \end{pmatrix}$$

The impact of error in the estimate of an effect is therefore limited to the subset of genotypes affected by that effect.

References

1. Poelwijk FJ, Krishna V, Ranganathan R (2016) The Context-Dependence of Mutations: A Linkage of Formalisms. *PLoS Comput Biol* 12(6):e1004771.

2. Weinberger ED (1991) Fourier and Taylor series on fitness landscapes. *Biol Cybern* 65(5):321–330.
3. Stadler PF (1996) Landscapes and their correlation functions. *J Math Chem* 20(1):1–45.
4. Weinreich DM, Lan Y, Wylie CS, Heckendorn RB (2013) Should evolutionary geneticists worry about higher-order epistasis. *Curr Opin Genet Dev* 23(6):700–707.
5. Stormo GD (2011) Maximally efficient modeling of DNA sequence motifs at all levels of complexity. *Genetics* 187(4):1219–1224.
6. Brookes DH, Aghazadeh A, Listgarten J (2022) On the sparsity of fitness functions and implications for learning. *Proc Natl Acad Sci USA* 119(1)

Appendix 1. Zero-mean property

The zero-mean property of reference-free effects is that for any site-combination (i_1, …, i_k), the mean of all effects that vary across a single site is zero:

$$e_{i_1,\dots,i_k}(\cdot, s_2, \dots, s_k) = e_{i_1,\dots,i_k}(s_1, \cdot, s_3, \dots, s_k) \dots = e_{i_1,\dots,i_k}(s_1, \dots, s_{k-1}, \cdot) = 0,$$

where (s_1, …, s_k) can be any state-combination. The zero-mean property is a defining feature of reference-free analysis, from which all of its useful properties proved in the next Appendices follow. We prove it by mathematical induction.

Recall that G_i^s is the set of all genotypes with state s in site i. $G_i^1, G_i^2, \dots, G_i^q$ are nonoverlapping sets whose union is G. Therefore, the summation $\Sigma_{\boldsymbol{g}\in G}$ is equivalent to $\Sigma_{s\in Q}\Sigma_{\boldsymbol{g}\in G_i^s}$. Then,

$$\begin{aligned}
e_i(\cdot) &= \frac{1}{q}\sum_{s\in Q} e_i(s) \\
&= \frac{1}{q}\sum_{s\in Q}[\langle y|G_i^s\rangle - \langle y|G\rangle] \\
&= \frac{1}{q}\sum_{s\in Q}\langle y|G_i^s\rangle - \langle y|G\rangle \\
&= \frac{1}{q}\sum_{s\in Q}\frac{1}{q^{n-1}}\sum_{\boldsymbol{g}\in G_i^s} y(\boldsymbol{g}) - \langle y|G\rangle \\
&= \frac{1}{q^n}\sum_{s\in Q}\sum_{\boldsymbol{g}\in G_i^s} y(\boldsymbol{g}) - \langle y|G\rangle \\
&= \frac{1}{q^n}\sum_{\boldsymbol{g}\in G} y(\boldsymbol{g}) - \langle y|G\rangle \\
&= 0.
\end{aligned}$$

We now show that if the zero-mean property holds for effects of order (k – 1), it also holds for effects of order k. Recall the definition

$$e_{i_1,\dots,i_k}(s_1, \dots, s_k) = e_{i_2,\dots,i_k}(s_2, \dots, s_k)|_{i_1}^{s_1} - e_{i_2,\dots,i_k}(s_2, \dots, s_k).$$

By the inductive hypothesis, the mean of the two (k – 1)-th-order terms on the right-hand side is zero across any site i_2, …, i_k. The mean of the k-th-order term on the left is therefore zero across any site i_2, …, i_k. Conditioning on a site other than i_1 shows that the mean across i_1 is also zero. This completes the mathematical induction.

Appendix 2. Generalized linear decomposition

Let $e_{i_1,\dots,i_k}$ be a function mapping *k*-tuples of states into real numbers. We refer to the following expression as the *k*-th-order generalized linear decomposition of genetic architecture:

$$y_k(\boldsymbol{g}) = e_0 + \sum_{i\in N} e_i(g_i) + \sum_{i_1<i_2\in N} e_{i_1,i_2}(g_{i_1}, g_{i_2}) + \cdots + \sum_{i_1<\cdots<i_k\in N} e_{i_1,\dots,i_k}\big(g_{i_1}, \dots, g_{i_k}\big). \quad \text{(A1)}$$

We showed that both reference-free and reference-based analyses can be represented as above with suitable choices of *e*. We now argue that any linear decomposition of genetic architecture can be represented as above with suitable choices of *e*. The validity of this statement depends on what a linear decomposition of genetic architecture is. Below we define it in the broadest possible sense and show that it has a form of Eq. (A1).

In the broadest sense, a linear decomposition of order zero is any function that approximates the phenotype of every genotype by a constant. It can thus be expressed as

$$y_0(\boldsymbol{g}) = e_0.$$

What is the broadest sense in which a function $y_1(\boldsymbol{g})$ is a first-order linear decomposition of genetic architecture? $y_1(\boldsymbol{g})$ should be able to use the information that $\boldsymbol{g}$ has the state g_i in site i and combine that information linearly across sites to determine its phenotype. It is not allowed, however, to use any information about what combination of states is found in what combination of sites. Any such function y_1 can be written as

$$y_1(\boldsymbol{g}) = \sum_j \lambda_j(\boldsymbol{g}),$$

where λ_j is a function that can distinguish whether $\boldsymbol{g}$ has a particular state in a particular site:

$$\lambda_j(\boldsymbol{g}) = \begin{cases} \alpha_j, & \boldsymbol{g} \in G_i^s \\ \beta_j, & \boldsymbol{g} \notin G_i^s \end{cases}$$

for some state *s,* some site *i*, and some real numbers α_j and β_j. The number of such functions that make up $y_1(\boldsymbol{g})$ is unlimited. It can be shown that for any such function y_1, we can find a constant e_0 and functions e_i such that

$$y_1(\boldsymbol{g}) = e_0 + \sum_{i\in N} e_i(g_i).$$

To prove this, we first sum all functions λ that distinguish whether $\boldsymbol{g} \in G_i^s$ for a given *s* and *i* and write the sum as

$$\lambda_i^s(\boldsymbol{g}) = \begin{cases} \alpha_i^s, & \boldsymbol{g} \in G_i^s \\ \beta_i^s, & \boldsymbol{g} \notin G_i^s. \end{cases}$$

y_1 can be written as

$$\begin{aligned} y_1(g) &= \sum_j \lambda_j(\boldsymbol{g}) \\ &= \sum_{i\in N}\sum_{s\in Q} \lambda_i^s(\boldsymbol{g}) \\ &= \sum_{i\in N}\sum_{s\in Q} [\lambda_i^s(\boldsymbol{g}) - \beta_i^s + \beta_i^s] \\ &= \sum_{i\in N}\sum_{s\in Q} [\lambda_i^s(\boldsymbol{g}) - \beta_i^s] + \sum_{i\in N}\sum_{s\in Q} \beta_i^s. \end{aligned}$$

Note that

$$\lambda_i^s(\boldsymbol{g}) - \beta_i^s = \begin{cases} \alpha_i^s - \beta_i^s, & \boldsymbol{g} \in G_i^s \\ 0, & \boldsymbol{g} \notin G_i^s \end{cases}$$

and thus

$$\sum_{s\in Q} [\lambda_i^s(\boldsymbol{g}) - \beta_i^s] = \alpha_i^{g_i} - \beta_i^{g_i}.$$

The following choice therefore completes the proof:

$$\begin{aligned} e_0 &= \sum_{i\in N}\sum_{s\in Q} \beta_i^s, \\ e_i(s) &= \alpha_i^s - \beta_i^s. \end{aligned}$$

Higher-order linear decompositions can be defined similarly. In the broadest sense, a second-order linear decomposition can use the information that $\boldsymbol{g}$ has the state g_i in site i and state-pair (g_{i_1}, g_{i_2}) in site-pair (i_1, i_2), but cannot use any information about higher-order combinations. Any such function y_2 can be written as

$$y_2(\boldsymbol{g}) = \sum_j \lambda_j(\boldsymbol{g}) + \sum_k \mu_k(\boldsymbol{g}),$$

where λ_j is defined as above and μ_k is a function that can distinguish whether $\boldsymbol{g}$ has a particular pair of states in a particular pair of sites:

$$\mu_k(\boldsymbol{g}) = \begin{cases} \gamma_k, & \boldsymbol{g} \in G_{i_1,i_2}^{s_1,s_2} \\ \delta_k, & \boldsymbol{g} \notin G_{i_1,i_2}^{s_1,s_2} \end{cases}$$

for some sites i_1 and i_2, some states s_1 and s_2, and some real numbers γ_k and δ_k. For any such function y_2, we can find a constant e_0 and functions e_i and e_{i_1,i_2} such that

$$y_2(\boldsymbol{g}) = e_0 + \sum_{i \in N} e_i(g_i) + \sum_{i_1 < i_2 \in N} e_{i_1,i_2}(g_{i_1}, g_{i_2}).$$

To prove this, we again sum all functions λ that distinguish whether $\boldsymbol{g} \in G_i^s$ for a given s and i and write the sum as

$$\lambda_i^s(\boldsymbol{g}) = \begin{cases} \alpha_i^s, & \boldsymbol{g} \in G_i^s \\ \beta_i^s, & \boldsymbol{g} \notin G_i^s \end{cases},$$

and similarly sum all functions μ that distinguish whether or not $\boldsymbol{g} \in G_{i_1,i_2}^{s_1,s_2}$ and write the sum as

$$\mu_{i_1,i_2}^{s_1,s_2}(\boldsymbol{g}) = \begin{cases} \gamma_{i_1,i_2}^{s_1,s_2}, & \boldsymbol{g} \in G_{i_1,i_2}^{s_1,s_2} \\ \delta_{i_1,i_2}^{s_1,s_2}, & \boldsymbol{g} \notin G_{i_1,i_2}^{s_1,s_2} \end{cases}.$$

Following a logic similar to above, we can choose

$$e_0 = \sum_{i \in N} \sum_{s \in Q} \beta_i^s + \sum_{i_1 < i_2 \in N} \sum_{s_1,s_2 \in Q} \beta_{i_1,i_2}^{s_1,s_2},$$
$$e_i(s) = \alpha_i^s - \beta_i^s,$$
$$e_{i_1,i_2}(s_1, s_2) = \gamma_{i_1,i_2}^{s_1,s_2} - \delta_{i_1,i_2}^{s_1,s_2}.$$

In general, a linear decomposition of order k in the broadest sense is any function that can use information about the combination of states in any set of up to k sites and combine that information linearly across site-combinations to determine the phenotype. A logic similar to above can show that any such function can be written as Eq. (A1).

Appendix 3. Optimal linear decomposition

Recall that any k-th-order linear decomposition of genetic architecture can be written as

$$y_k(\boldsymbol{g}) = e_0 + \sum_{i\in N} e_i(g_i) + \sum_{i_1<i_2\in N} e_{i_1,i_2}(g_{i_1}, g_{i_2}) + \cdots + \sum_{i_1<\cdots<i_k\in N} e_{i_1,\ldots,i_k}(g_{i_1}, \ldots, g_{i_k}).$$

Here we show that the reference-free definition of effects minimizes the sum of squared error

$$\epsilon_G = \sum_{\boldsymbol{g}\in G} [y(\boldsymbol{g}) - y_k(\boldsymbol{g})]^2,$$

for any genetic architecture at any value of k. We prove this by showing that the partial derivative of ε_G with respect to a term is zero when that term is defined by the reference-free definition. Using e to denote a term,

$$\begin{aligned}\frac{\partial \epsilon_G}{\partial e} &= \frac{\partial}{\partial e}\sum_{\boldsymbol{g}\in G} [y(\boldsymbol{g}) - y_k(\boldsymbol{g})]^2 \\ &= \sum_{\boldsymbol{g}\in G} \frac{\partial}{\partial e}[y(\boldsymbol{g}) - y_k(\boldsymbol{g})]^2 \\ &= \sum_{\boldsymbol{g}\in G} -2[y(\boldsymbol{g}) - y_k(\boldsymbol{g})]\frac{\partial}{\partial e} y_k(\boldsymbol{g}).\end{aligned}$$

The derivative of $y_k(\boldsymbol{g})$ with respect to e is 1 if $y_k(\boldsymbol{g})$ involves e and 0 otherwise. For example, for $e = e_i(s)$, the derivative of $y_k(\boldsymbol{g})$ is 1 for all $\boldsymbol{g} \in G_i^s$ and 0 for all $\boldsymbol{g} \notin G_i^s$. For $e = e_{i_1,\ldots,i_k}(s_1, \ldots, s_k)$, let G_e denote the set $G_{i_1,\ldots,s_k}^{s_1,\ldots,s_k}$. Then,

$$\frac{\partial}{\partial e} y_k(\boldsymbol{g}) = \begin{cases} 1, & \boldsymbol{g} \in G_e \\ 0, & \boldsymbol{g} \notin G_e \end{cases}.$$

The partial derivative of ε_G is then

$$\begin{aligned}\frac{\partial \epsilon_G}{\partial e} &= \sum_{\boldsymbol{g}\in G} -2[y(\boldsymbol{g}) - y_k(\boldsymbol{g})]\frac{\partial}{\partial e} y_k(\boldsymbol{g}) \\ &= -2\sum_{\boldsymbol{g}\in G_e} [y(\boldsymbol{g}) - y_k(\boldsymbol{g})] \\ &= -2q^{n-O(e)}[\langle y|G_e\rangle - \langle y_k|G_e\rangle],\end{aligned}$$

where $O(e)$ is the order of e. The partial derivative is zero when

$$\langle y|G_e\rangle = \langle y_k|G_e\rangle.$$

In other words, an optimal linear decomposition of order k is that which accurately predicts the average phenotype of any subset of genotypes defined by fixing up to k sites. We defined reference-free effects to achieve just that! To formally show this, we prove the following property of reference-free analysis:

Lemma 1. Recall that $e_{i_1,\dots,i_k}(g_{i_1},\dots,g_{i_k})$ denotes the k-th-order effect of state-combination $(g_{i_1},\dots,g_{i_k})$ in site-combination (i_1, …, i_k). Due to the zero-mean property, the mean of $e_{i_1,\dots,i_k}(g_{i_1},\dots,g_{i_k})$ across all genotypes is zero. We now calculate the mean across subsets of genotypes defined by fixing the states at some sites. Consider the set $G_{j_1,\dots,j_l}^{s_{j_1},\dots,s_{j_l}}$, which comprises all genotypes with states $s_{j_1},\dots,s_{j_l}$ in sites j_1, …, j_l. Lemma 1 claims that

$$\left\langle e_{i_1,\dots,i_k} \middle| G_{j_1,\dots,j_l}^{s_{j_1},\dots,s_{j_l}} \right\rangle = 0$$

unless (i_1, …, i_k) is a subset of (j_1, …, j_l), in which case

$$\left\langle e_{i_1,\dots,i_k} \middle| G_{j_1,\dots,j_l}^{s_{j_1},\dots,s_{j_l}} \right\rangle = e_{i_1,\dots,i_k}(s_{i_1},\dots,s_{i_k}).$$

This follows from the zero-mean property: if any site among i_1, …, i_k lies outside the set (j_1, …, j_l), the above summation involves summing across all q states in that site.

Before proving Lemma 1, let us see how it helps proving the optimality of reference-free analysis. For any effect $e = e_{j_1,\dots,j_l}(s_{j_1},\dots,s_{j_l})$, where $l \le k$,

$$\begin{aligned}\langle y_k|G_e\rangle &= \frac{1}{q^{n-l}} \sum_{g\in G_{j_1,\dots,j_l}^{s_{j_1},\dots,s_{j_l}}} y_k(\boldsymbol{g}) \\ &= \frac{1}{q^{n-l}} \sum_{g\in G_{j_1,\dots,j_l}^{s_{j_1},\dots,s_{j_l}}} \left[e_0 + \sum_{i\in N} e_i(g_i) + \sum_{i_1<i_2\in N} e_{i_1,i_2}(g_{i_1},g_{i_2}) + \cdots + \sum_{i_1<\cdots<i_k\in N} e_{i_1,\dots,i_k}(g_{i_1},\dots,g_{i_k}) \right].\end{aligned}$$

Due to Lemma 1, the sum for any site-combination that is not a subset of (j_1, …, j_l) is zero. Therefore, using L to denote the set {1, 2, …, l},

$$\langle y_k|G_e\rangle = e_0 + \sum_{\alpha\in L} e_{j_\alpha}(s_{j_\alpha}) + \sum_{\alpha_1<\alpha_2\in L} e_{j_{\alpha_1},j_{\alpha_2}}(s_{j_{\alpha_1}},s_{j_{\alpha_2}}) + \cdots + \sum_{\alpha_1<\cdots<\alpha_{l-1}\in L} e_{j_{\alpha_1},\dots,j_{\alpha_{l-1}}}\left(s_{j_{\alpha_1}},\dots,s_{j_{\alpha_{l-1}}}\right) + e_{j_1,\dots,j_l}(s_{j_1},\dots,s_{j_l}).$$

This, by the definition of reference-free decomposition, equals $\left\langle y \middle| G_{j_1,\dots,j_l}^{s_{j_1},\dots,s_{j_l}} \right\rangle = \langle y|G_e\rangle$:

$$\left\langle y \middle| G_{j_1,\dots,j_l}^{s_{j_1},\dots,s_{j_l}} \right\rangle = \left\langle y \middle| G_{j_1,\dots,j_l}^{s_{j_1},\dots,s_{j_l}} \right\rangle_{(l-1)} + e_{j_1,\dots,j_l}(s_{j_1},\dots,s_{j_l}),$$

where

$$\left\langle y \middle| G_{j_1,\dots,j_l}^{s_{j_1},\dots,s_{j_l}} \right\rangle_{(l-1)} = e_0 + \sum_{\alpha\in L} e_{j_\alpha}(s_{j_\alpha}) + \sum_{\alpha_1<\alpha_2\in L} e_{j_{\alpha_1},j_{\alpha_2}}(s_{j_{\alpha_1}},s_{j_{\alpha_2}}) + \cdots + \sum_{\alpha_1<\cdots<\alpha_{l-1}\in L} e_{j_{\alpha_1},\dots,j_{\alpha_{l-1}}}\left(s_{j_{\alpha_1}},\dots,s_{j_{\alpha_{l-1}}}\right).$$

Proof of Lemma 1.

Consider first the case when $(i_1,\dots,i_k) \subseteq (j_1,\dots,j_l)$. Across the set $G_{j_1,\dots,j_l}^{s_{j_1},\dots,s_{j_l}}$, which consists only of genotypes with states $s_{j_1},\dots,s_{j_l}$ in sites $j_1,\dots,j_l$, the term $e_{i_1,\dots,i_k}(g_{i_1},\dots,g_{i_k})$ is a constant, $e_{i_1,\dots,i_k}(s_{i_1},\dots,s_{i_k})$. This proves the second case of the lemma.

Let us now assume that *m* sites in $(i_1,\dots,i_k)$ are outside $(j_1,\dots,j_l)$. We make our notation flexible so that the order of sites can be permuted: for example, $e_{i_1,i_2}(s_1,s_2) = e_{i_2,i_1}(s_2,s_1)$. We can then write

$$(i_1,\dots,i_k) = (x_1,\dots,x_{k-m},y_1,\dots,y_m),$$

where $x_1,\dots,x_{k-m}$ are inside $(j_1,\dots,j_l)$ and $y_1,\dots,y_m$ are outside. $G_{j_1,\dots,j_l}^{s_{j_1},\dots,s_{j_l}}$ can be partitioned as

$$G_{j_1,\dots,j_l}^{s_{j_1},\dots,s_{j_l}} = \bigcup_{t_1,\dots,t_m\in Q} G_{j_1,\dots,j_l,y_1,\dots,y_m}^{s_{j_1},\dots,s_{j_l},t_1,\dots,t_m}.$$

Therefore, the summation $\Sigma_{g\in G_{j_1,\dots,j_l}^{s_{j_1},\dots,s_{j_l}}}$ equals $\Sigma_{t_1,\dots,t_m\in Q}\Sigma_{g\in G_{j_1,\dots,j_l,y_1,\dots,y_m}^{s_{j_1},\dots,s_{j_l},t_1,\dots,t_m}}$. It follows then

$$\begin{aligned}
\left\langle e_{i_1,\dots,i_k} \middle| G_{j_1,\dots,j_l}^{s_{j_1},\dots,s_{j_l}} \right\rangle &= \frac{1}{q^{n-l}} \sum_{g\in G_{j_1,\dots,j_l}^{s_{j_1},\dots,s_{j_l}}} e_{i_1,\dots,i_k}\left(g_{i_1},\dots,g_{i_k}\right) \\
&= \frac{1}{q^{n-l}} \sum_{t_1,\dots,t_m\in Q} \sum_{g\in G_{j_1,\dots,j_l,y_1,\dots,y_m}^{s_{j_1},\dots,s_{j_l},t_1,\dots,t_m}} e_{x_1,\dots,x_{k-m},y_1,\dots,y_m}\left(s_{x_1},\dots,s_{x_{k-m}},t_1,\dots,t_m\right) \\
&= \frac{q^l}{q^{n-l}} \sum_{t_1,\dots,t_m\in Q} e_{x_1,\dots,x_{k-m},y_1,\dots,y_m}\left(s_{x_1},\dots,s_{x_{k-m}},t_1,\dots,t_m\right) \\
&= 0.
\end{aligned}$$

The last equality follows from the zero-mean property.

Appendix 4. Variance partition

To help derive the variance partition formula, we use $Var\left(e_{i_1,\dots,i_k}|Q^k\right)$ to denote the variance of all k-th-order effects at the site-combination (i_1, …, i_k).

$$Var\left(e_{i_1,\dots,i_k}|Q^k\right) = \frac{1}{q^k}\sum_{s_1,\dots,s_k\in Q}\left[e_{i_1,\dots,i_k}(s_1,\dots,s_k) - \left\langle e_{i_1,\dots,i_k}|Q^k\right\rangle\right]^2$$
$$= \frac{1}{q^k}\sum_{s_1,\dots,s_k\in Q} e_{i_1,\dots,i_k}(s_1,\dots,s_k)^2,$$

where the last equality follows from the zero-mean property. We show that the total phenotypic variance

$$V = Var(y|G) = \frac{1}{q^n}\sum_{\boldsymbol{g}\in G}[y(\boldsymbol{g}) - \langle y|G\rangle]^2$$

can be decomposed into the variance of reference-free effects at each site-combination:

$$V = \sum_{i\in N} Var(e_i|Q) + \sum_{i_1<i_2\in N} Var\left(e_{i_1,i_2}|Q^2\right) + \cdots + \sum_{i_1<\cdots<i_k\in N} Var\left(e_{i_1,\dots,i_k}|Q^k\right) + \cdots + Var\left(e_{1,\dots,n}|Q^n\right).$$

This is equivalent to the variance partition formula

$$V = \sum_{e\neq e_0}\frac{e^2}{q^{O(e)}}.$$

Recall that for any genotype $\boldsymbol{g} = (g_1,\dots,g_n)$,

$$y(\boldsymbol{g}) = e_0 + \sum_{i\in N} e_i(g_i) + \cdots + \sum_{i_1<\cdots<i_k\in N} e_{i_1,\dots,i_k}\left(g_{i_1},\dots,g_{i_k}\right) + \cdots + e_{i_1,\dots,i_n}(g_1,\dots,g_n).$$

Substituting this expression into the definition of V, we obtain

$$V = \frac{1}{q^n}\sum_{\boldsymbol{g}\in G}\left[\sum_{i\in N} e_i(g_i) + \cdots + \sum_{i_1<\cdots<i_k\in N} e_{i_1,\dots,i_k}\left(g_{i_1},\dots,g_{i_k}\right) + \cdots + e_{i_1,i_2,\dots,i_n}(g_1,g_2,\dots,g_n)\right]^2. \quad \text{(A2)}$$

To simplify this equation, we prove the following lemma.

Lemma 2. For any two distinct site-combinations $(i_1,\dots,i_k) \neq (j_1,\dots,j_l)$,

$$\frac{1}{q^n}\sum_{g\in G} e_{i_1,\dots,i_k}\left(g_{i_1},\dots,g_{i_k}\right) e_{j_1,\dots,j_l}\left(g_{j_1},\dots,g_{j_l}\right)=0.$$

Before proving Lemma 2, let us check how it helps simplify Eq. (A2). Recall that $(\sum_i x_i)^2 = \sum_i x_i^2 + \sum_{i,j} x_i x_j$. Under Lemma 2, Eq. (A2) simplifies to

$$V=\frac{1}{q^n}\sum_{g\in G}\left[\sum_{i\in N} e_i(g_i)^2+\cdots+\sum_{i_1<\cdots<i_k\in N} e_{i_1,\dots,i_k}\left(g_{i_1},\dots,g_{i_k}\right)^2+\cdots+e_{i_1,i_2,\dots,i_n}(g_1,g_2,\dots,g_n)^2\right].$$

The set *G* can be expressed as the union $G=\bigcup_{s_1,\dots,s_k\in Q} G_{i_1,\dots,i_k}^{s_1,\dots,s_k}$ for any state-combination (s_1, …, s_k) in site-combination (i_1, …, i_k). Therefore,

$$\begin{aligned}\frac{1}{q^n}\sum_{g\in G} e_{i_1,\dots,i_k}\left(g_{i_1},\dots,g_{i_k}\right)^2 &= \frac{1}{q^n}\sum_{s_1,\dots,s_k\in Q}\ \sum_{g\in G_{i_1,\dots,i_k}^{s_1,\dots,s_k}} e_{i_1,\dots,i_k}\left(g_{i_1},\dots,g_{i_k}\right)^2\\ &= \frac{1}{q^n}\sum_{s_1,\dots,s_k\in Q}\ \sum_{g\in G_{i_1,\dots,i_k}^{s_1,\dots,s_k}} e_{i_1,\dots,i_k}(s_1,\dots,s_k)^2\\ &= \frac{1}{q^n}\sum_{s_1,\dots,s_k\in Q} q^{n-k} e_{i_1,\dots,i_k}(s_1,\dots,s_k)^2\\ &= \frac{1}{q^k}\sum_{s_1,\dots,s_k\in Q} e_{i_1,\dots,i_k}(s_1,\dots,s_k)^2\\ &= Var\left(e_{i_1,\dots,i_k}|Q^k\right).\end{aligned}$$

This then proves the variance partition formula.

Proof of Lemma 2.

We make our notation flexible so that the order of sites can be permuted: for example, $e_{i_1,i_2}(s_1,s_2)=e_{i_2,i_1}(s_2,s_1)$. Assume *m* sites are shared between $(i_1,\dots,i_k)$ and $(j_1,\dots,j_l)$. We can write

$$\begin{aligned}(i_1,\dots,i_k)&=(a_1,\dots,a_m,x_1,\dots x_{k-m}),\\ (j_1,\dots,j_l)&=(a_1,\dots,a_m,y_1,\dots y_{l-m}),\end{aligned}$$

where $(x_1,\dots x_{k-m})\cap(y_1,\dots y_{l-m})=\emptyset$. We partition *G* as follows:

$$G=\bigcup_{s_1,\dots,s_m\in Q}\ \bigcup_{u_1,\dots,u_{k-m}\in Q}\ \bigcup_{v_1,\dots,v_{l-m}\in Q} G_{a_1,\dots,a_m,x_1,\dots,x_{k-m},y_1,\dots,y_{l-m}}^{s_1,\dots,s_m,u_1,\dots,u_{k-m},v_1,\dots,v_{l-m}}.$$

Then,

$$
\begin{aligned}
&\frac{1}{q^n}\sum_{\boldsymbol{g}\in G} e_{i_1,\dots,i_k}\left(g_{i_1},\dots,g_{i_k}\right) e_{j_1,\dots,j_l}\left(g_{j_1},\dots,g_{j_l}\right) \\
&= \frac{q^{n-(k+l-m)}}{q^n} \sum_{s_1,\dots,s_m\in Q}\ \sum_{u_1,\dots,u_{k-m}\in Q}\ \sum_{v_1,\dots,v_{l-m}\in Q} e_{i_1\dots i_k}(s_1,\dots,s_m,u_1,\dots,u_{k-m}) e_{j_1\dots j_l}(s_1,\dots,s_m,v_1,\dots,v_{l-m}) \\
&= \frac{1}{q^{k+l-m}} \sum_{s_1,\dots,s_m\in Q}\ \sum_{u_1,\dots,u_{k-m}\in Q} e_{i_1\dots i_k}(s_1,\dots,s_m,u_1,\dots,u_{k-m}) \sum_{v_1,\dots,v_{l-m}\in Q} e_{j_1\dots j_l}(s_1,\dots,s_m,v_1,\dots,v_{l-m}) \\
&= 0.
\end{aligned}
$$

where the last equality follows from the zero-mean property. A similar proof can be constructed for when no site is shared between $(i_1,\dots,i_k)$ and $(j_1,\dots,j_l)$.

Appendix 5. Robustness to measurement noise

Our goal is to calculate reference-free effects for an unstructured genetic architecture in which each phenotype is an independent sample from a noise distribution of zero mean and variance ω. The exact value of each effect depends on the particular instantiation of the sampling process. We are interested in the variance of each effect across the possible instantiations. Let Ω denote the set of all possible instantiations. We aim to compute

$$Var(e|\Omega),$$

which, by the definition of variance, equals

$$\langle e^2|\Omega\rangle - \langle e|\Omega\rangle^2 = \langle e^2|\Omega\rangle,$$

where the last equality follows because the expected value of any phenotype and therefore any effect is zero. We prove the following formula by mathematical induction:

$$Var(e|\Omega) = \frac{(q-1)^{O(e)}}{q^n}\omega. \quad \text{(A3)}$$

Consider the intercept:

$$\begin{aligned} Var(e_0|\Omega) &= Var\left[\frac{1}{q^n}\textstyle\sum_{\boldsymbol{g}\in G} y(\boldsymbol{g})\,\middle|\Omega\right] \\ &= \frac{1}{q^{2n}}\sum_{\boldsymbol{g}\in G} Var[y(\boldsymbol{g})|\Omega] \\ &= \frac{1}{q^{2n}}\sum_{\boldsymbol{g}\in G} \omega \\ &= \frac{\omega}{q^n}. \end{aligned}$$

This is Eq. (A3) for the case when $O(e)$ equals zero. Let us now assume that Eq. (A3) holds for effects of order $(k-1)$. Recall the definition

$$e_{i_1,\dots,i_k}(s_1,\dots,s_k) = e_{i_2,\dots,i_k}(s_2,\dots,s_k)|_{i_1}^{s_1} - e_{i_2,\dots,i_k}(s_2,\dots,s_k),$$

in which an effect of order k is a function of effects of order $(k-1)$. We prove that for any $j \neq i_1,\dots,i_k$,

$$e_{i_1,\dots,i_k}(s_1,\dots,s_k) = \frac{1}{q}\sum_{t\in Q} e_{i_1,\dots,i_k}(s_1,\dots,s_k)|_j^t. \quad \text{(A4)}$$

That is, an effect of order *k* for the complete space *G* is the average of the same effect calculated for the subspaces defined by conditioning on any site outside the *k* focal sites. Before proving Eq. (A4), let us see how it helps us:

$$
\begin{aligned}
e_{i_1,\ldots,i_k}(s_1,\ldots,s_k) &= e_{i_2,\ldots,i_k}(s_2,\ldots,s_k)|_{i_1}^{s_1} - e_{i_2,\ldots,i_k}(s_2,\ldots,s_k) \\
&= e_{i_2,\ldots,i_k}(s_2,\ldots,s_k)|_{i_1}^{s_1} - \frac{1}{q}\sum_{t\in Q} e_{i_2,\ldots,i_k}(s_2,\ldots,s_k)|_{i_1}^{t} \\
&= \frac{q-1}{q} e_{i_2,\ldots,i_k}(s_2,\ldots,s_k)|_{i_1}^{s_1} - \frac{1}{q}\sum_{t\in Q\setminus s_1} e_{i_2,\ldots,i_k}(s_2,\ldots,s_k)|_{i_1}^{t}.
\end{aligned}
$$

Note that the q terms $e_{i_2,\ldots,i_k}(s_2,\ldots,s_k)|_{i_1}^{t}$, $t = 1, \ldots, q$, are probabilistically independent of each other because they involve genotypes from disjoint sub-spaces. Furthermore,

$$
\begin{aligned}
Var\left[e_{i_2,\ldots,i_k}(s_2,\ldots,s_k)\middle|\Omega\right] &= Var\left[\frac{1}{q}\textstyle\sum_{t\in Q} e_{i_2,\ldots,i_k}(s_2,\ldots,s_k)|_{i_1}^{t}\middle|\Omega\right] \\
&= \frac{1}{q} Var\left[e_{i_2,\ldots,i_k}(s_2,\ldots,s_k)|_{i_1}^{t'}\middle|\Omega\right],
\end{aligned}
$$

where t' in the last term can be any one of the q states. This implies that

$$
Var\left[e_{i_2,\ldots,i_k}(s_2,\ldots,s_k)|_{i_1}^{t'}\middle|\Omega\right] = q \times Var\left[e_{i_2,\ldots,i_k}(s_2,\ldots,s_k)\middle|\Omega\right] = \frac{q(q-1)^{k-1}}{q^n}\omega.
$$

From the aforementioned probabilistic independence, it follows that

$$
\begin{aligned}
Var\left[e_{i_1,\ldots,i_k}(s_1,\ldots,s_k)\middle|\Omega\right] &= \left[\left(\frac{q-1}{q}\right)^2 + \frac{q-1}{q^2}\right] \times \frac{q(q-1)^{k-1}}{q^n}\omega \\
&= \frac{(q-1)^k}{q^n}\omega.
\end{aligned}
$$

We now turn to proving Eq. (A4). We can rewrite $e_i(s)$ purely in terms of average phenotypes:

$$
e_i(s) = \langle y|G_i^s\rangle - \langle y|G\rangle.
$$

Similarly,

$$
e_{i_1,i_2}(s_1,s_2) = \left\langle y\middle|G_{i_1,i_2}^{s_1,s_2}\right\rangle - \left\langle y\middle|G_{i_1}^{s_1}\right\rangle - \left\langle y\middle|G_{i_2}^{s_2}\right\rangle + \langle y|G\rangle.
$$

In general,

$$
e_{i_1,\ldots,i_k}(s_1,\ldots,s_k) = \left\langle y\middle|G_{i_1,\ldots,i_k}^{s_1,\ldots,s_k}\right\rangle - \sum_{\alpha_1<\cdots<\alpha_{k-1}\in K} \left\langle y\middle|G_{i_{\alpha_1},\ldots,i_{\alpha_{k-l}}}^{s_{\alpha_1},\ldots,s_{\alpha_{k-l}}}\right\rangle + \sum_{\alpha_1<\cdots<\alpha_{k-2}\in K} \left\langle y\middle|G_{i_{\alpha_1},\ldots,i_{\alpha_{k-2}}}^{s_{\alpha_1},\ldots,s_{\alpha_{k-2}}}\right\rangle - \cdots,
$$

where K is the set 1, …, k. More compactly,

$$e_{i_1,\dots,i_k}(s_1,\dots,s_k) = \sum_{0\le l\le k}(-1)^{k-l}\sum_{\alpha_1<\cdots<\alpha_l\in K}\left\langle y \middle| G_{i_{\alpha_1},\dots,i_{\alpha_l}}^{s_{\alpha_1},\dots,s_{\alpha_l}}\right\rangle.$$

What is important here is that $e_{i_1,\dots,i_k}(s_1,\dots,s_k)$ is a linear combination of the average phenotype of every possible subset of G defined by fixing the states at one or more of the k sites i_1, …, i_k. Any such average phenotype can be decomposed by fixing the state at another site $j \neq i_1,\dots,i_k$. For example,

$$\left\langle y \middle| G_{i_1}^{s_1}\right\rangle = \frac{1}{q}\sum_{t\in Q}\left\langle y \middle| G_{i_1,j}^{s_1,t}\right\rangle.$$

Therefore,

$$\begin{aligned} e_{i_1,\dots,i_k}(s_1,\dots,s_k) &= \frac{1}{q}\sum_{t\in Q}\sum_{0\le l\le k}(-1)^{k-l}\sum_{\alpha_1<\cdots<\alpha_l\in K}\left\langle y \middle| G_{i_{\alpha_1},\dots,i_{\alpha_l},j}^{s_{\alpha_1},\dots,s_{\alpha_l},t}\right\rangle \\ &= \frac{1}{q}\sum_{t\in Q} e_{i_1,\dots,i_k}(s_1,\dots,s_k)|_{G_j^t}. \end{aligned}$$

Appendix 6. Unbiased estimation by regression

We show that unbiased estimates of reference-free effects can be obtained through a two-step procedure. We first solve the optimization

$$\hat{y}_k = \text{argmin} \sum_{\boldsymbol{g} \in G^*} [y(\boldsymbol{g}) - y_k(\boldsymbol{g})]^2. \tag{A5}$$

Because of the degeneracy of generalized linear decomposition, there are many solutions to this optimization. We choose any solution and enforce the zero-mean property by changing its terms without altering the predicted phenotype. We show that this normalization is always possible. We then show that $\hat{y}_k$ thus obtained is an unbiased estimate of reference-free decomposition.

We reformulate Eq. (A5) as a standard regression. Let y be a vector of sampled phenotypes and β a vector of effects to infer. We write

$$y = X\beta + \epsilon, \tag{A6}$$

where X is the design matrix specifying how the effects map to phenotypes. The error ε is the sum of all unmodeled higher-order effects and measurement noise. The solution to this regression is not unique: X is a singular matrix because of the degeneracy of generalized linear decomposition. We can make X non-singular by building in the zero-mean property. For example, we can eliminate the column of X corresponding to $e_i(q)$ by coding it as

$$e_i(q) = -\sum_{t \in Q \setminus q} e_i(t).$$

Similarly, we can eliminate the column of X corresponding to $e_{i_1,i_2}(s_1, q)$ by coding it as

$$e_{i_1,i_2}(s_1, q) = -\sum_{t \in Q \setminus q} e_{i_1,i_2}(s_1, t).$$

In general, every term involving state q in any site can be eliminated by coding it as a linear combination of terms involving states 1 to $(q - 1)$ in accordance with the zero-mean property. The design matrix thus obtained is non-singular and can be used to infer for all terms only involving states 1 to $(q - 1)$. Estimates for terms involving state q can be calculated post-hoc using the zero-mean property. This proves the existence of a solution $\hat{y}_k$ whose terms satisfy the zero-mean property.

We now show that the expected value of the error ε in Eq. (A6) is zero across randomly sampled genotypes. Since the design matrix is non-singular and the errors are unbiased, by the Gauss-Markov theorem the regression estimates for terms containing states 1 to $(q - 1)$ are unbiased. The post-hoc estimates for terms containing state q are also unbiased because they are linear combinations of terms containing states 1 to $(q - 1)$.

In a linear decomposition of order *k*, the error for a genotype $\boldsymbol{g}$ is given by

$$\epsilon(\boldsymbol{g}) = \sum_{i_1<\cdots<i_{k+1}\in N} e_{i_1,\dots,i_{k+1}}\left(g_{i_1},\dots,g_{i_{k+1}}\right) + \cdots + e_{1,\dots,n}(g_1,\dots,g_n).$$

(Measurement noise is not shown because it does not affect the expected value of ε.) Since genotypes are randomly sampled, it suffices to show that the expected value of ϵ is zero across all genotypes. We prove a stronger result:

$$\begin{aligned}
\langle e_{i_1,\dots,i_k} | G\rangle &= \frac{1}{q^n}\sum_{\boldsymbol{g}\in G} e_{i_1,\dots,i_k}(g_1,\dots,g_k) \\
&= \frac{1}{q^n}\sum_{s_1,\dots,s_k\in Q}\ \sum_{\boldsymbol{g}\in G_{i_1,\dots,i_k}^{s_1,\dots,s_k}} e_{i_1,\dots,i_k}(g_1,\dots,g_k) \\
&= \frac{1}{q^n}\sum_{s_1,\dots,s_k\in Q}\ \sum_{\boldsymbol{g}\in G_{i_1,\dots,i_k}^{s_1,\dots,s_k}} e_{i_1,\dots,i_k}(s_1,\dots,s_k) \\
&= \frac{1}{q^k}\sum_{s_1,\dots,s_k\in Q} e_{i_1,\dots,i_k}(s_1,\dots,s_k) \\
&= 0.
\end{aligned}$$

It follows that $\langle \epsilon | G\rangle = 0$.

We now show how the zero-mean property can be enforced post hoc. Consider enforcing the zero-mean property on a first-order linear decomposition without altering the predicted phenotypes (hereafter called "normalizing"). To normalize the terms for site *i*, we first subtract from each term the mean of all terms at site *i*:

$$\delta_i(s) = e_i(s) - e_i(\cdot) \Longrightarrow \delta_i(\cdot) = 0.$$

Using $\delta_i(s)$ in place of $e_i(s)$ alters the predicted phenotype of every genotype by $-e_i(\cdot)$. This can be corrected by adding $e_i(\cdot)$ to the intercept. Overall, the following modifications normalize any first-order linear decomposition:

$$\begin{aligned}
\delta_i(s) &= e_i(s) - e_i(\cdot), \\
\delta_0 &= e_0 + \sum_{i\in N} e_i(\cdot).
\end{aligned}$$

Similarly, the following modifications normalize any second-order linear decomposition:

$$\begin{aligned}
\delta_{i_1,i_2}(s_1,s_2) &= e_{i_1,i_2}(s_1,s_2) - e_{i_1,i_2}(\cdot,s_2) - e_{i_1,i_2}(s_1,\cdot) + e_{i_1,i_2}(\cdot,\cdot), \\
\delta_i(s) &= [e_i(s) - e_i(\cdot)] + \sum_{j\in N\setminus i}\left[e_{i,j}(s,\cdot) - e_{i,j}(\cdot,\cdot)\right],
\end{aligned}$$

$$\delta_0 = e_0 + \sum_{i \in N} e_i(\cdot) + \sum_{i_1 < i_2 \in N} e_{i_1,i_2}(\cdot,\cdot).$$

For any third-order linear decomposition:

$$\begin{aligned}\delta_{i_1,i_2,i_3}&(s_1,s_2,s_3) \\ &= e_{i_1,i_2,i_3}(s_1,s_2,s_3) - \left[e_{i_1,i_2,i_3}(\cdot,s_2,s_3) + e_{i_1,i_2,i_3}(s_1,\cdot,s_3) + e_{i_1,i_2,i_3}(s_1,s_2,\cdot)\right] \\ &\quad + \left[e_{i_1,i_2,i_3}(\cdot,\cdot,s_3) + e_{i_1,i_2,i_3}(\cdot,s_2,\cdot) + e_{i_1,i_2,i_3}(s_1,\cdot,\cdot)\right] - e_{i_1,i_2,i_3}(\cdot,\cdot,\cdot),\end{aligned}$$

$$\begin{aligned}\delta_{i_1.i_2}(s_1,s_2) &= e_{i_1,i_2}(s_1,s_2) - \left[e_{i_1,i_2}(\cdot,s_2) + e_{i_1,i_2}(s_1,\cdot)\right] + e_{i_1,i_2}(\cdot,\cdot) \\ &\quad + \sum_{i_3 \in N \setminus i_1,i_2} \left[e_{i_1,i_2,i_3}(s_1,s_2,\cdot) - e_{i_1,i_2,i_3}(\cdot,s_2,\cdot) - e_{i_1,i_2,i_3}(s_1,\cdot,\cdot) + e_{i_1,i_2,i_3}(\cdot,\cdot,\cdot)\right],\end{aligned}$$

$$\delta_i(s) = [e_i(s) - e_i(\cdot)] + \sum_{j \in N \setminus i} \left[e_{i,j}(s,\cdot) - \epsilon_{ij}(\cdot,\cdot)\right] + \sum_{j<k \in N \setminus i} \left[e_{i,j,k}(s,\cdot,\cdot) - \epsilon_{i,j,k}(\cdot,\cdot,\cdot)\right],$$

$$\delta_0 = \epsilon_0 + \sum_{i \in N} e_i(\cdot) + \sum_{i_1 < i_2 \in N} e_{i_1,i_2}(\cdot,\cdot) + \sum_{i_1 < i_2 < i_3 \in N} e_{i_1,i_2,i_3}(\cdot,\cdot,\cdot).$$

Directly applying these normalization formulae is cumbersome for high orders. We provide a simple algorithm. First, we normalize the highest-order terms (order k) without correcting for the altered phenotypes. This can be done by Eq. (A7) shown below. Then, let y_k denote the phenotype predicted by the original linear decomposition and z_k the phenotypic contribution of the normalized k-th-order terms. We must modify the lower-order terms so that their total phenotypic contribution is $y_k - z_k$. This can be done by using regression to find a linear model of order $(k-1)$ whose predicted phenotype is $y_k - z_k$. Such a linear model exists because post hoc enforcement of zero-mean property is always possible. Terms of order $(k-1)$ can then be normalized and the same regression procedure can be used to correct for the altered phenotypes by changing the terms of order up to $(k-2)$.

To show how terms of order k can be normalized, we introduce a notation: $e_{i_1,\dots,i_k}(s_1,\dots,s_k)_{j_1,\dots j_l}$ denotes the mean of $e_{i_1,\dots,i_k}(s_1,\dots,s_k)$ across sites $j_1, \dots, j_l$. For example,

$$\begin{aligned}e_{i_1,\dots,i_k}(s_1,\dots,s_k)_{i_1} &= e_{i_1,\dots,i_k}(\cdot,s_2,\dots,s_k), \\ e_{i_1,\dots,i_k}(s_1,\dots,s_k)_{i_1,i_2} &= e_{i_1,\dots,i_k}(\cdot,\cdot,s_3,\dots,s_k).\end{aligned}$$

Normalization of a second-order term can be restated as

$$\delta_{i_1,i_2}(s_1,s_2) = e_{i_1,i_2}(s_1,s_2) - \sum_{\alpha \in \{1,2\}} e_{i_1,i_2}(s_1,s_2)_{i_\alpha} + e_{i_1,i_2}(s_1,s_2)_{i_1,i_2}.$$

Denoting the set $\{1, \dots, k\}$ by K, terms of order k can be normalized by

$$\delta_{i_1,\dots,i_k}(s_1,\dots,s_k) = e_{i_1,\dots,i_k}(s_1,\dots,s_k) + \sum_{l\in K}(-1)^l \sum_{\alpha_1<\cdots<\alpha_l\in K} e_{i_1,\dots,i_k}(s_1,\dots,s_k)_{i_{\alpha_1},\dots,i_{\alpha_l}}. \quad \text{(A7)}$$
